# From lactation to malignancy: A comparison between healthy and cancerous breast gland at single‐cell resolution reveals new issues for tumorigenesis

**DOI:** 10.1002/1873-3468.70162

**Published:** 2025-09-08

**Authors:** Pietro Ancona, Carlo M. Bergamini, Carlo Ferrari, Stefano Volinia, Nicoletta Bianchi

**Affiliations:** ^1^ Department of Translational Medicine University of Ferrara Italy; ^2^ Department of Neuroscience and Rehabilitation University of Ferrara Italy; ^3^ Department of Clinical and Molecular Sciences (DISCLIMO) School of Medicine and Surgery, Polytechnic University of Marche (UNIVPM) Ancona Italy; ^4^ Biological and Chemical Research Centre (CNBCh) University of Warsaw Poland

**Keywords:** breast cancer, lactocytes, MALAT1, mammary gland, prognostic markers, single‐cell RNA sequencing, transcription factors, transcriptomics

## Abstract

This study, based on datasets from healthy tissues, lactating mammary epithelial cells, and breast cancer phenotypes, investigates mammary gland pathophysiology at single‐cell resolution to identify key regulators in breast cancer development and to gain a deeper understanding of its biology and heterogeneity. We suggest that antileukoproteinase (SLPI) has prognostic value associated with metastasis in basal breast cancers. Our analysis highlights the similarity between triple‐negative breast cancer cells and mature luminal lactocytes, which share active regulons (SOX2, MTHFD1, POU4F3, and ZNF32), suggesting conserved molecular mechanisms. Among the differences, the absence of MALAT1 and NEAT1 lncRNAs in lactocytes correlates with loss of six transcription factors (EP300, ELF1, E2F3, BDP1, HOXC10, and KLF6). These findings provide insights into breast cancer and suggest new therapeutic targets.

## Graphical abstract

Single‐cell RNA sequencing reveals an opposite role of SLPI in basal tumors based on metastatic spread, along with shared activation of specific regulons in cancer cells and mature luminal lactocytes, as well as downregulation of MALAT1 and NEAT1 in the latter. These findings improve the understanding of cancer and provide novel opportunities for new treatments.

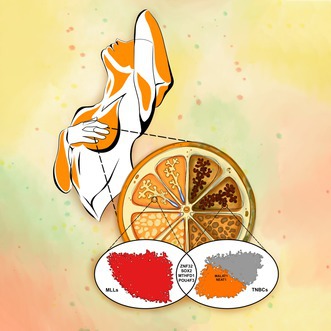

## Abbreviations

AUC, area under the curve

BrCa, breast cancer

cl, cluster

CNV, copy number variation

DCs, dendritic cells

DEA, differential expression analysis

DPT, diffusion pseudotime

ECM, extracellular matrix

ER, estrogen receptor

FDR, false discovery rate

GEPIA2, gene expression profile interactive analysis 2

GRN, gene regulatory network

HER2, human epidermal growth factor

HR, hazard ratio

LCs, luminal cells

LDCs, luminal differentiating cells

LGR4, Leucine‐Rich Repeat Containing G Protein‐Coupled Receptor 4

lncRNAs, long noncoding RNAs

log2FC, log2 fold change

LPs, luminal progenitors

MLLs, mature luminal lactocytes

MYO myoepithelial

NPBCs, nonproliferative basal cells

OS, overall survival

PAGA, partition‐based graph abstraction

PBCs, proliferative basal cells

PR, progesterone receptor

Rb, retinoblastoma

RFS, relapse‐free survival

scRNA‐seq, single‐cell RNA‐sequencing

SNPs, single‐nucleotide polymorphism

TFs, transcription factors

TNBC triple‐negative breast cancer

UMAP, uniform manifold approximation and projection

The mammary gland is a complex organ that comprises different cell types, which precisely collaborate to maintain breast homeostasis. The basal layer is mainly formed by myoepithelial (MYO) cells, whose role is both to provide mechanical support and contractile functions to the outer cell layers [1]. The functional compartment of the mammary gland is formed by the alveolar and lobular cells derived from luminal progenitors (LPs), which differentiate into mature milk‐producing luminal cells, the lactocytes. The development of the breast gland begins during embryogenesis with the formation of the mammary ridge, from which mammary buds sprout around the twelfth week of gestation. These buds penetrate the underlying mesenchyme, giving rise to primary ducts. Postnatally, the gland remains relatively quiescent until puberty, when hormonal changes, particularly increased estrogen and progesterone levels, stimulate ductal elongation, branching, and lobuloalveolar differentiation. During pregnancy, further proliferation and differentiation occur, preparing the gland for lactation, driven by prolactin, progesterone, and oxytocin. After weaning, the gland undergoes involution, characterized by apoptosis and remodeling of the extracellular matrix (ECM). In adulthood, cyclic hormonal changes during the menstrual cycle induce fluctuations in glandular tissue, affecting proliferation and regression [2, 3].

Abnormal hormonal signaling, particularly involving estrogen and progesterone receptors (ER and PR), can drive proliferation in hormone‐responsive tissues, contributing to tumor development, along with genetic and epigenetic alterations disrupting the tightly regulated processes of cell proliferation, apoptosis, and differentiation. Additionally, mutations in oncogenes (e.g., *PIK3CA* and *MYC*) and tumor suppressor genes (e.g., *BRCA1*, *BRCA2*, and *TP53*) can lead to uncontrolled cell growth and malignancy. The tumor microenvironment, including stromal cells, immune cells, and ECM components, also plays a critical role in promoting tumor progression, invasion, and metastasis. The heterogeneity of breast cancer (BrCa) reflects variations in these genetic, epigenetic, and microenvironmental factors, leading to distinct subtypes with differing prognoses and treatment responses.

Despite new therapeutic approaches being developed to counter BrCa, such as immunotherapy and molecular targeting, and an increase in positive outcomes being registered, still a large cohort of patients die because of BrCa, especially those with a triple‐negative phenotype. For this reason, the understanding of BrCa biology needs to be expanded.

The two main transformations involving the mammary gland are lactation and tumorigenesis. These two physiological and pathological processes are extremely different from each other, even though they might share common programs; therefore, finely deciphering their similarities and divergences could shed light on the underlying mechanisms of tumorigenesis. The few studies conducted in this direction evidenced a reduced risk of developing both breast and ovarian cancer for women who have breastfed [4, 5]. These studies have the limitation that no one has yet considered the great heterogeneity of BrCa and adequately explored their phenotypic differences. Triple‐negative breast cancer (TNBC) is the most aggressive phenotype, showing the poorest survival and the highest recurrence rate. This subtype arises from the basal layer of the breast, and no commonly used drugs are directed against specific molecular targets. Interestingly, TNBC could also present lactating features, normally associated with gestation and breastfeeding [6] and epidemiological studies correlated this type of tumor with a negative prognosis depending on the breastfeeding duration [7, 8]. Although an association between breastfeeding and BrCa development has been found, these studies do not delve into the molecular mechanisms for their better characterization.

New OMICS technologies, such as single‐cell RNA sequencing (scRNA‐seq), represent a huge breakthrough in this perspective. The scRNA‐seq technology is a great resource to investigate the complex scenario of the breast gland development in a new and innovative way, with a resolution that was impossible to achieve with the canonical RNA sequencing approach, and which can help fill the gap between the epidemiological knowledge and the molecular understanding of BrCa.

This manuscript aimed to compare the physiological and pathological aspects of the mammary gland at single‐cell resolution, highlighting shared behavior and key regulatory players involved in the rise and growth of the most diagnosed cancer within the female population [9].

## Materials and methods

### Samples used in scRNA‐seq analysis

We used data obtained from 20 samples of scRNA‐seq downloaded from GEO (GSE161529, GSE245601) and Array Express (E‐MTAB‐9841) databases. These breast samples include tissue biopsies from different BrCa phenotypes: human epidermal growth factor (HER2)‐enriched (*n* = 4), ER^+^ (*n* = 4), and TNBC (*n* = 4), healthy tissues (*n* = 4), and epithelial cells isolated from milk in breastfeeding (*n* = 4). The number of cells obtained from each sample is reported in Table S1.

### Quality controls and raw data processing

Raw data quality was assessed with MultiQC [10], and fastq files were aligned to the GRCh38 genome reference using the Genomic Cell Ranger v8.0.1 software [11]. CellBender v0.3.1 (https://github.com/broadinstitute/CellBender) has been employed to filter out empty droplets [12]. After the first steps of quality check, files were loaded in the Python environment and processed using Scanpy v1.10.3 [13]. All samples were merged into a single anndata object, comprising 143,727 single‐cell RNA profiles (Table S1).

To rule out technical artifacts and errors, a further quality check protocol was applied to the samples. Initially, doublets were removed using the integrated Scrublet package of Scanpy (https://github.com/swolock/scrublet) [14]. Then, all cells expressing less than 1000 total counts were cleared out. A modified Z‐score approach was employed to statistically identify the outliers. Data points deviating from the median by more than *n*‐median absolute deviations were excluded, and cells expressing more than 5% of mitochondrial counts were removed.

The raw counts were normalized to a total count of 10.000 per cell and then log‐transformed using a logarithm plus one (log1p). After principal component analysis, the dataset was integrated using the Harmony Scanpy integrated function (https://github.com/immunogenomics/harmony) [15], which performed well with this type of data. To identify the different clusters, the Leiden algorithm was employed at a resolution of 0.5, while we used the Wilcoxon rank‐sum test to pinpoint cluster‐specific markers. Finally, we manually annotated the clusters using the most upregulated genes coding for the markers associated with specific cell types.

### Gene Regulatory Network analysis

To analyze transcription factors (TFs) and regulon activation, we performed a Gene Regulatory Network (GRN) analysis using only differentially expressed TFs. We integrated the results derived from two analysis methods to reduce the probability of false positives. In the first analysis, all significantly ranked genes of scRNAseq profiles derived from the Wilcoxon rank‐sum test of each cluster were extracted and merged with the list of TFs (https://resources.aertslab.org/cistarget/motif2tf/motifs‐v9‐nr.hgnc‐m0.001‐o0.0.tbl, accessed the 11 January 2025). In the second analysis, we used a pseudo‐bulk approach with the aid of the decoupler package v1.9.1 (https://decoupler‐py.readthedocs.io/en/latest/index.html). These two lists of differentially expressed TFs were further narrowed, considering only those with a false discovery rate (FDR) < 0.05 and log_2_FC > 2 or < −2.

This list of significantly altered TFs was used as input for pySCENIC package v0.12.1 (https://github.com/aertslab/pySCENIC) [16]. This allows the prediction of TFs–target interaction and then infers GRN and activation/deactivation of transcriptional cascades in the different cell types.

### Copy number variation inferring analysis

To better explore the results, we loaded the anndata file in RStudio (Posit Software, PBC, Boston, MA, USA), converted it into a Seurat Object using the Schard package (https://github.com/cellgeni/schard), and employed the copyKAT software v1.1.0 (https://github.com/navinlabcode/copykat) [17] to determine the chromosomal status (aneuploid/diploid) of each cell.

### Reference mapping

To further investigate the phenotype of our basal cancer cells (NPBCs and PBCs), we mapped them onto the *Mammary Gland Development* atlas (https://github.com/MarioniLab/MammaryGland, accessed on 12 July 2025). We downloaded the sequencing and annotation data from GEO (accession GSE106273) and loaded them into Python as an *anndata* object. We first assessed data quality using the same filtering approach described above in the “Quality controls and raw data processing” section. Since our data originate from human samples while the atlas is based on murine cells, we converted murine gene names to their corresponding human orthologs using the *mousipy* package (v0.1.7) (https://doi.org/10.5281/zenodo.15631326, accessed on 12 July 2025).

We then identified the genes shared between the reference *anndata* and our basal cell *anndata* and filtered both datasets to retain only commonly expressed genes. To project our cells into the UMAP space of the reference atlas, we used the ingest function from the *scanpy* package. This tool enables the integration of embeddings and annotations from a query *anndata* into a reference dataset by projecting onto a dimensionality reduction model, the uniform manifold approximation and projection (UMAP), using a K‐nearest neighbors classifier for label transfer, and the *UMAP* package for embedding alignment. To visualize the results, we plotted both the query (basal cancer) cells and the reference cells within the same UMAP space.

## Results and discussion

### Cellular heterogeneity assessment and cluster profiling

**Fig. 1:**
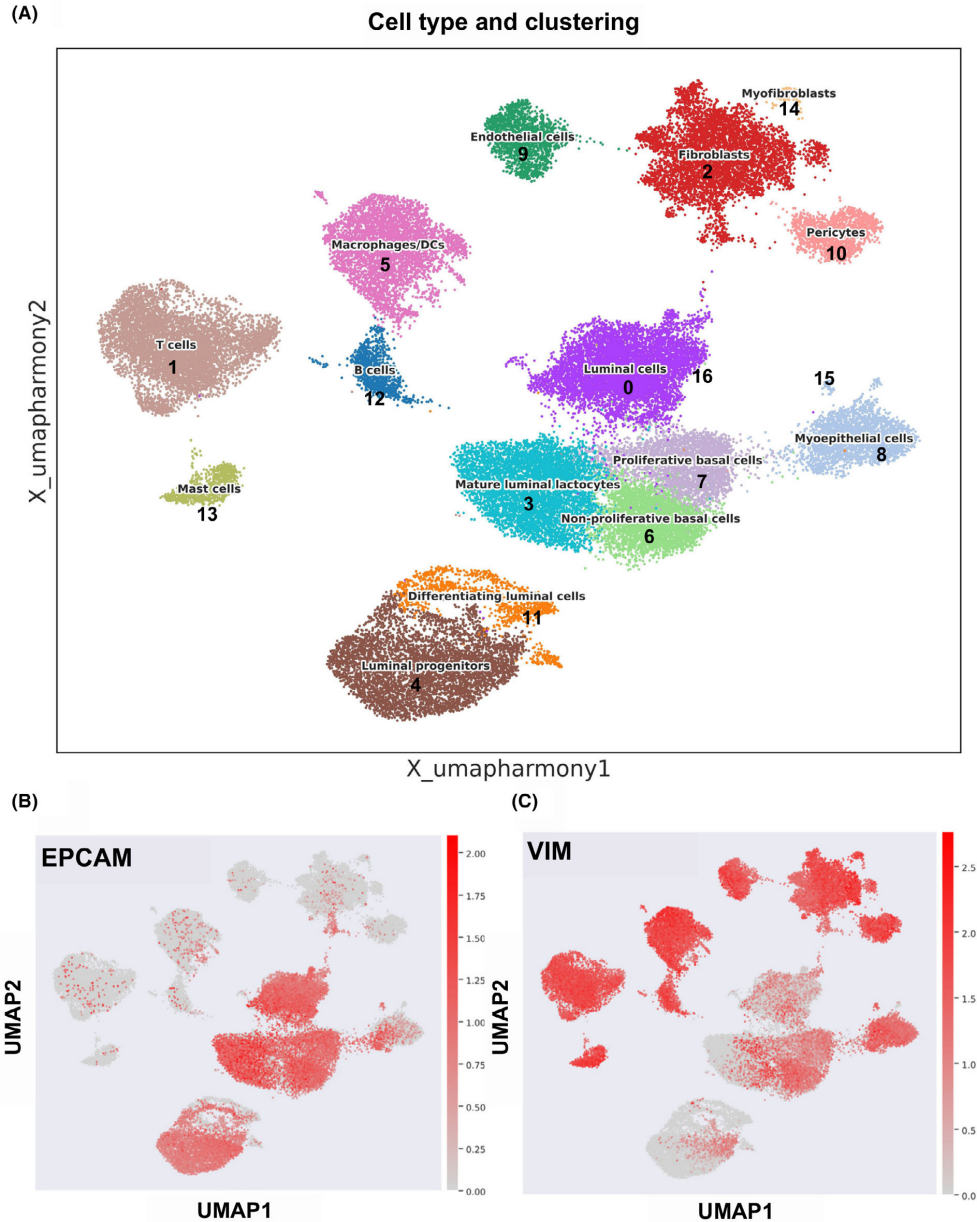
Uniform Manifold Approximation and Projection plot (UMAP) of single‐cell RNA (scRNA) profiles. UMAP representation of different clusters using the Leiden clustering at 0.5 resolution (A). The different clusters have both the numeral identifier according to the clustering and the annotated cell type. The *EPCAM* (B) and *VIM* (C) expression is depicted. The colored bars on the right side describe the expression levels of the considered markers. Red spots correspond to the strong expression of the marker, while gray spots correspond to absent expression.

In this study, we used 143.727 single‐cell RNA profiles obtained from databases (GSE161529, GSE245601, E‐MTAB‐9841) derived from biopsies of BrCas (HER2‐enriched, ER^+^, TNBC), healthy tissues, and epithelial cells isolated from breast milk of lactating women and reported in Table S1. Data were processed to exclude errors and technical artifacts, as detailed in the Materials and Methods section. Since samples originating from distinct datasets are likely to be affected by batch effects due to factors such as different operators or sequencing protocols, we assessed their relevance prior to downstream analyses in order to apply appropriate corrections. As shown in the UMAP in Fig. S1a, a clear batch effect was evident, characterized by an almost complete separation of clusters according to color, which corresponds to the dataset of origin. To address this issue, we applied the scanpy.external.pp.harmony integrate function. The results, shown in the UMAP in Fig. S1b, displayed a strong overlap within immune and stromal cell clusters (upper part of the panel), while only partial overlap was observed in the epithelial compartment (lower part of the panel). This pattern reflects the intrinsic heterogeneity of normal and tumor tissues, which should be preserved and further explored through subsequent analyses. Therefore, we deliberately avoided modifying the integration parameters in order to prevent masking biologically relevant differences between samples, which are crucial for a robust interpretation of the data. The resulting 50.976 single‐cell profiles were clustered by the Leiden algorithm at 0.5 resolution as the best fitting to obtain a well‐defined separation of cell types. The analysis revealed 17 clusters (cl), although cl‐16 and cl‐0 represent the same cell type, as well as cl‐15 and cl‐8 (Fig. 1A). Cells have been annotated in clusters based on the highest‐ranked expressed genes and on established cell‐type‐specific markers. As the first step, we aimed to discriminate between epithelial and mesenchymal groups employing *EPCAM* (Fig. 1B) to identify epithelial clusters and *VIM* (Fig. 1C) as a marker for the mesenchymal compartment.

The assignment of clusters has been confirmed by the expression of additional markers associated with specific cell types, and stromal or epithelial features (Fig. S2 and Fig. S3). Also, immune system‐related clusters were identified as follows: cl‐1 represented T cells/NK, cl‐12 B cells, cl‐5 dendritic cells (DCs)/macrophages, and cl‐13 mast cells. All stromal contributions were identified by the following markers (Fig. S2): cl‐2 expressing *DCN*, *COL3A1*, and *LUM* corresponding to fibroblast, cl‐9 with *PECAM1* and *ADGRL4* identified the endothelial cells, cl‐10 with *ABCC9* and *RGS5* the pericytes. The epithelial cell compartment was the most complex, containing contributions from different samples: BrCa phenotypes, healthy breast tissues, and epithelial cells isolated from the milk of lactating women. Therefore, epithelial clusters have been annotated using a set of highest‐ranked markers (Fig. S3): cl‐0 and cl‐16 contained cells derived predominantly from ER^+^, HER2‐enriched tumors, and healthy tissues. Although cancerous and normal cells usually display different gene expression signatures, they also exhibit common features, mostly related to the lineage they are committed to. In this case, cl‐0 and cl‐16 showed high levels of *CDH1* (E‐cadherin), *FOXA1*, and *KRT8*, suggesting a luminal phenotype, and we annotated them as luminal cells (LCs), coherently with the common clustering, while we found two clusters derived from TNBCs (cl‐6 and cl‐7) showing basal features and expressing *KRT5, KRT14*, and *KRT17* markers, coherently with their TNBC origin, but divided into two different clusters, suggesting at least partial divergent gene expression. This is supported by the upregulation of proliferation‐related genes in cl‐7 (Fig. S4a–c), typical of proliferative basal cells (PBCs), while cl‐6 represents mainly nonproliferative basal cells (NPBCs). Indeed, cl‐7 showed the highest number of cells in the G2/M phase compared with the other clusters (Fig. S4d). Another cluster containing TNBCs was cl‐8, including healthy cells, and presenting MYO features. In cl‐8, we detected the expression of epithelial *KRT5* and *KRT17* markers at highest gene rank scores, associated with basal lineage, pinpointing a commitment of cl‐8 cells to represent an inner layer of the breast gland. Moreover, among the most upregulated genes, we found *ACTA2*, *MYLK*, and *TP63* suggesting a MYO phenotype [18]. In physiological conditions, the MYO cells are located beneath the luminal epithelial cells, surrounding the milk‐producing units (alveoli and ducts), giving both support and contractile features to the mammary gland. In cancerous tissue, the disruption of this supporting layer is a landmark of invasive lesions. Also, the role of MYO cells is to maintain the breast tissue development to the basal membrane, while under tumorigenic conditions, they could contribute to the growth of the tumoral mass secreting growth‐related and neo‐angiogenic factors [1].

Cells derived from the lactating breast gland (cl‐3, cl‐4, and cl‐11) exhibited a gene expression signature different from that of other luminal‐derived cells (cl‐16 and cl‐0), confirming the different nature laying underneath the two developing processes of the breast gland, lactation and neoplastic transformation. Based on the cluster‐specific markers, we inferred their identity. In cl‐3, we found both expression of luminal markers and upregulation of genes related to milk production (*CSN3*, *LALBA*, *CSN1S1*, *LTF*, and *BTN1A1*), at higher levels in comparison with that of cl‐4 and cl‐11, which also are milk‐derived epithelial cells. For this reason, we pointed them to be mature luminal lactocytes (MLLs), as shown in Fig. S3. Indeed, cl‐4 showed a progenitor‐like profile with upregulation of LP markers such as *TLR2* [19], *SOX10* [20], *PROM1*, and *KRT15* [21]. Concerning cl‐11, the gene expression of these cells falls between the MLLs and the LPs, suggesting a more heterogeneous cell composition, also supported by the upregulation of differentiation markers pointing to luminal differentiating cells (LDCs) (Fig. S3).

Since the goal of our analysis is to delve into the epithelial cell fate during lactation and neoplastic growth, we restricted only to epithelial clusters, including luminal, basal, and MYO cells.

### Tracing the origin of MYO cells in the cl‐8 lineage

**Fig. 2:**
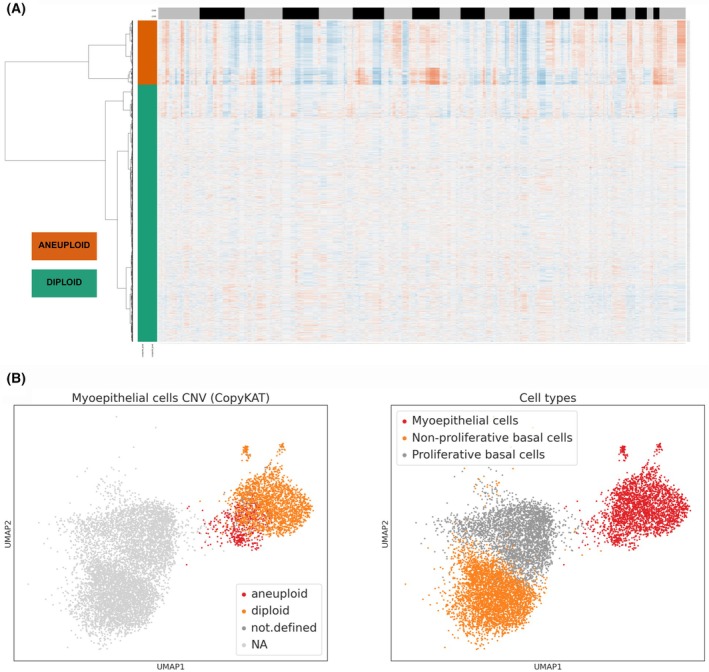
CopyKAT copy number variation (CNV) and Uniform Manifold Approximation and Projection plot (UMAP) plot of myoepithelial (MYO) cells. (A) CopyKAT CNV heatmap of MYO cells. The heatmap shows inferred whole‐genome CNVs for individual MYO cells. Chromosomes are arranged sequentially along the X‐axis, and each row on the Y‐axis represents a single cell. Cells grouped by the orange sidebar are computationally inferred as aneuploid (malignant), while those grouped by the green sidebar are inferred as diploid (nonmalignant/normal). Color intensity within the heatmap reflects the copy number status: blue indicates deletions, and red indicates chromosomal amplifications. (B) UMAP plots of MYO cells. Left panel, inferred ploidy status of MYO cells from triple‐negative breast cancer (TNBC) and healthy donors; right panel, cell type annotations shown for reference.

Considering the presence of a mixed population of cl‐8 (MYO cells) in both cancerous and healthy samples, we investigated whether these cells were diploid or aneuploid. In the former case, they could represent healthy “contaminants” from the tumor microenvironment, whereas in the latter case, their origin might be linked to transdifferentiation from tumor cells. Analysis of copy number variations (CNVs) using the CopyKAT algorithm revealed that a proportion of the cells within cl‐8 were indeed aneuploid (Fig. 2A). We further assessed the ploidy status on the UMAP (Fig. 2B), where aneuploid cells were clearly localized on the left side, in close proximity to the two lower clusters representing basal‐like cancer cells.

**Fig. 3:**
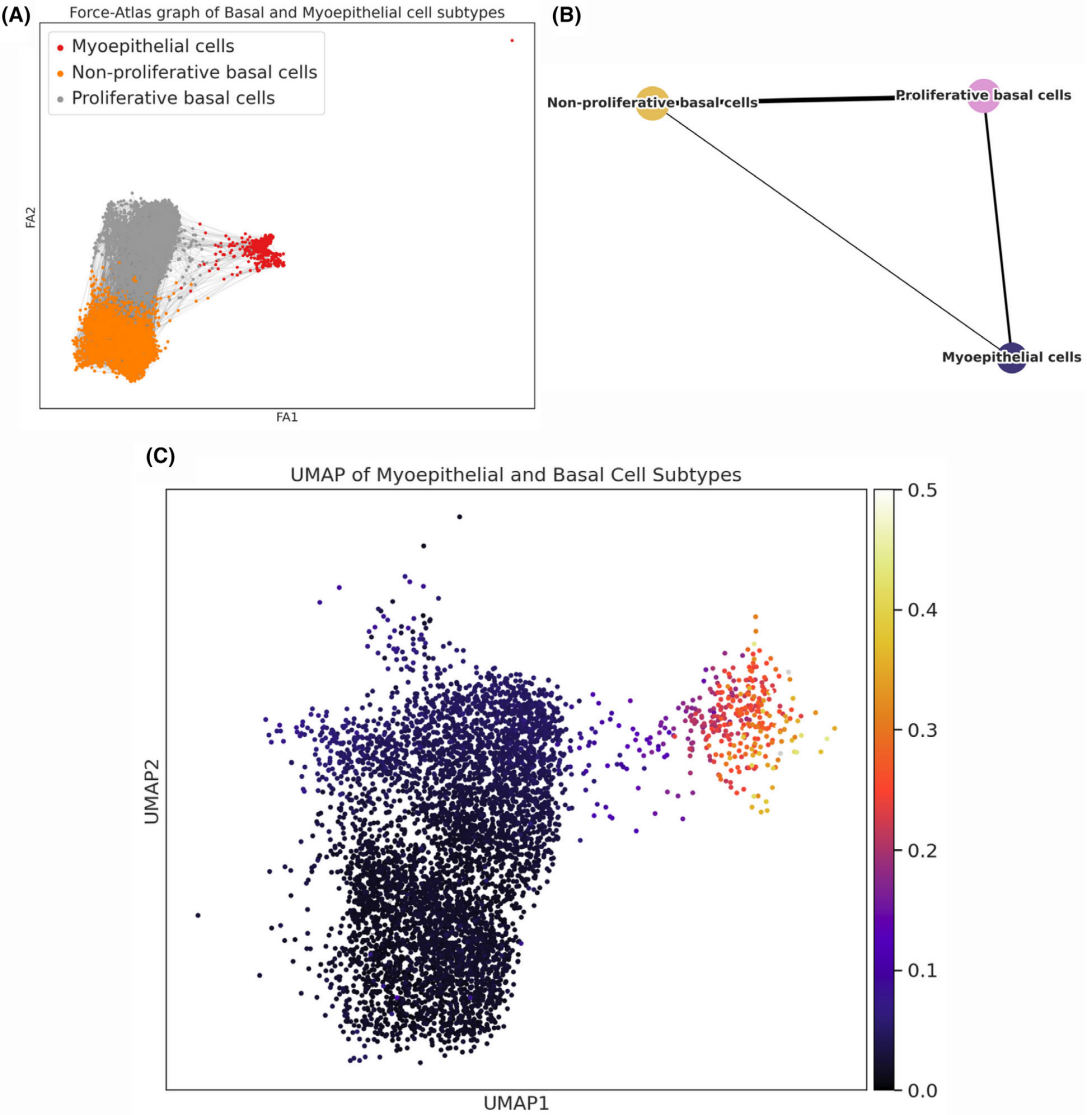
Single‐cell transcriptomic and Uniform Manifold Approximation and Projection (UMAP) embedding, inferred connections and pseudotime. (A) Force‐directed graph visualization of single cells. Each node represents an individual cell, colored according to its assigned cell type: gray, proliferative basal cancer (PBC); orange, nonproliferative basal cancer (NPBC); red, myoepithelial (MYO)‐like. The layout, derived from a k‐nearest neighbors' graph and optimized using the ForceAtlas2 algorithm, places transcriptionally similar cells in closer proximity. Gray edges indicate transcriptional similarities (connections within the k‐nearest neighbors' graph) between individual cells, highlighting the continuum and potential transitions among the distinct cell statuses. (B) each node (dot) represents an aggregated cell status cluster (PBC, NPBC, MYO). Edges connecting the clusters denote significant inferred relationships or transitions between these states. Darker edge intensity reflects stronger connections or higher transition probabilities between the two connected clusters, indicating greater transcriptional similarity and shared cellular trajectories. (C) UMAP embedding colored by inferred pseudotime. This UMAP displays individual cells embedded in a two‐dimensional space according to their transcriptional similarity. Each cell is colored based on its inferred pseudotime value, with a continuous color gradient ranging from dark purple/black (representing early states, pseudotime ≈ 0.0) to bright yellow/white (representing later or more differentiated states, pseudotime ≈ 0.5). This trajectory reflects the progressive transcriptional changes occurring during the observed cellular differentiation or transdifferentiation process.

Concerning the origin from basal tumor cells, we performed a pseudotime analysis to investigate the continuum of cellular status, positioning along the trajectory representing their relative status within the progression of the underlying biological process. We isolated cells belonging to the PBC, NPBC, and MYO clusters, all derived from samples labeled as TNBC. Using the integrated tools in Scanpy, namely partition‐based graph abstraction (PAGA, where edge weights represent the confidence of connections) and diffusion pseudotime (DPT, which infers cell progression based on geodesic distances along the graph), we investigated the differentiation trajectory of basal cancer cells. The results revealed a continuous differentiation path starting from nonproliferative basal cells, progressing through the proliferative basal status, and ultimately transitioning into MYO‐like cells. Indeed, in Fig. 3A, we can observe the trajectory connections, with a clear predominance of links between PBC and MYO cells; in Fig. 3B, the overall interactions among the three clusters highlight a strong connection between NPBC and PBC, and between PBC and MYO cells, but a weaker one between NPBC and MYO cells, suggesting a two‐step differentiation process: from NPBC to PBC and from PBC to MYO. In Fig. 3C, the UMAP on the left shows pseudotime values, confirming the derivation of MYO cells from basal cells, as indicated by the color scale (black represents the cells defined as the “starting point,” while MYO cells display higher scores, indicating their origin from a differentiated status derived from the starting point).

Considering the heterogeneous nature of the cells within the MYO cluster, we analyzed their gene expression patterns based on well‐established mammary gland markers [22]. Data are reported in Fig. S5. All basal compartment markers (KRT5, KRT14, and KRT17) were expressed in both the tumor‐derived and healthy MYO components, indicating a shared basal lineage. Regarding markers specific to differentiated MYO cells (ACTA2, MYLK, PDPN, and CXCL14), they were expressed, albeit at variable levels, in both tumor‐associated and healthy MYO cells. The hypothesis that transdifferentiation originated from mammary epithelial cells is further supported by the lower expression of MYO progenitor markers (TP63, BPTF, and NRG1) in tumor‐associated “MYO‐like” cells, while these markers were present in MYO cells from healthy tissue. Finally, we assessed the expression of markers associated with a mammary stem cell‐like (MaSC‐like) status, typically found in mammary stem cells (CD24 and CLDN4); these markers were expressed exclusively in tumor MYO cells and not in their healthy counterparts, providing additional support for the transdifferentiation hypothesis of tumor‐derived MYO‐like cells.

In addition, a differential gene expression analysis was performed using the Wilcoxon rank‐sum test to compare the transcriptome of tumor‐associated MYO‐like cells with that of healthy MYO cells (Fig. S6). This analysis revealed a significant upregulation in tumor cells of several genes known to be associated with mammary carcinogenesis, such as CD24, as well as glycolytic enzymes such as TPI1 and PKM, indicative of a metabolically active condition. TAGLN2, an actin‐binding protein involved in cell motility and contractility, was also upregulated. Furthermore, several members of the S100 family (S100P, S100A4, S100A9, and S100A14), which are linked to calcium flux and cellular contractility, were upregulated. Additional markers associated with ECM interaction and remodeling (CLDN4, TM4SF1, TACSTD2, PERP, and CAPG) were also significantly upregulated in tumor MYO cells.

These findings strongly support the hypothesis that the mixed population observed in cl‐8 does not result from contamination by normal MYO cells but instead reflects a process of MYO transdifferentiation.

### Context‐dependent prognostic value of *SLPI* in metastatic and nonmetastatic basal BrCa

**Fig. 4:**
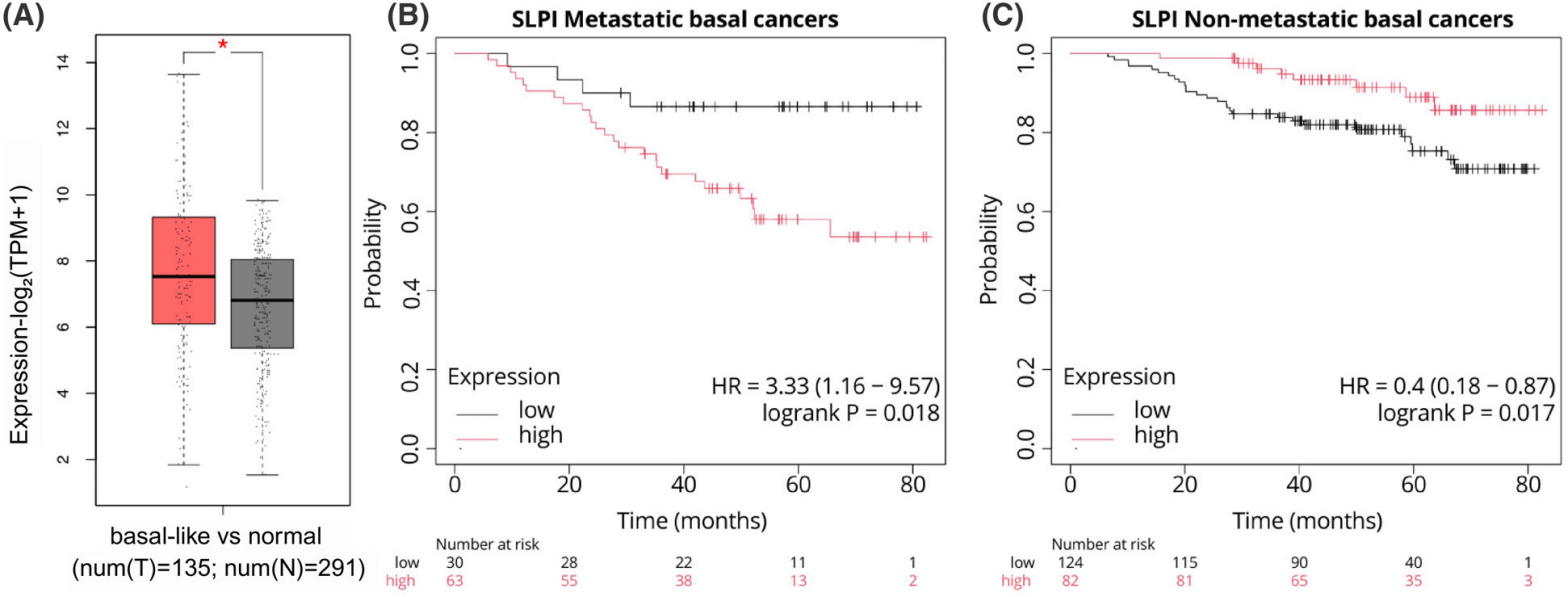
Overall survival (OS) plots and expression of *SLPI*. (A) the boxplot of *SLPI* expression in basal‐like tumors (red box) and normal (gray box) clinical samples (*P*‐value < 0.05). Differential expression was assessed by one‐way ANOVA using disease state (Tumor vs. Normal) as the grouping variable. The horizontal line at the top and at the bottom of the boxes represents the maximum and minimum values of the category (whiskers) in –log_2_(TPM + 1) (transcript per million). The red asterisks indicate significant differences between the compared pairs (*P*‐value < 0.05). Clinical data were retrieved from The Cancer Genome Atlas (TCGA) and Genotype‐Tissue Expression (GTEx) databases. The OS of patients affected by metastatic (B) and nonmetastatic basal (C) Breast cancers (BrCas) is reported in relation to *SLPI* expression. In the *X* axis, the time in months, while in the *Y* axis, the probability of OS indicated in decimals (0–1).

Alongside basal markers, a gene among the most upregulated (expressed at the same level of the KRT5 and KRT17 cytokeratins) in the two basal clusters (cl‐6, NPBCs, and cl‐7, PBCs) is *SLPI*, which encodes a protease inhibitor protecting epithelia from serine proteases. Indeed, this protein is expressed in secreting epithelial cells, and according to the Human Protein Atlas (proteinatlas.org accessed on 11 January 2025) [23], *SLPI* is enriched in the lung‐, bronchus‐, and salivary gland‐related cell types; however, it is also expressed in other tissues, including breast in a milder fashion. Although SLPI involvement was evidenced in different cancers, its role in neoplastic growth is controversial. We employed the database Gene Expression Profile Interactive Analysis (GEPIA2) [24] evidencing an upregulation of *SLPI* (log_2_FC = 1.43; *P*‐value < 0.01) in basal‐like BrCa (*n* = 135) compared with normal tissue (*n* = 291) (Fig. 4A).

In TNBCs, SLPI plays a role in metastatic spreading, physically interacting with and inhibiting retinoblastoma (Rb) protein tumor suppressor [25, 26]. To check the relevance of *SLPI* upregulation, we employed TGCA and GTEx data. Since *SLPI* is upregulated in basal BrCa compared with non‐basal ones [27], we analyzed the overall survival (OS), differentiating specimens based on whether a tumor had or had not the metastatic spread [28]. Using specific parameters, such as hazard ratio (HR) and *P*‐value of logrank (logrank*P*), we compared the survival distributions between two groups. The HR value indicates the relative risk of an event occurring in the group of patients with metastatic events compared to the nonmetastatic. Values > 1 mean that the subjects have a higher risk. The significance of HR is measured using the logrank test obtaining the logrank*P* value, which indicates significance when < 0.05. Interestingly, we found *SLPI* to be an unfavorable prognostic factor in metastasis‐carrying patients (HR = 3.33, logrank*P* = 0.018, Fig. 4B), in agreement with a previously published study on metastatic TNBC [25], while in nonmetastatic basal BrCa, *SLPI* acted oppositely, representing a positive prognostic marker (HR = 0.4, logrank*P* = 0.017, Fig. 4C). We hypothesize that *SLPI* exerts protective behavior in non‐invasive tumors; conversely, in metastatic cancer, it contributes to a worse prognosis.

Our hypothesis is supported by a study conducted by Munn and Garkavtsev [26], who identified this serine protease inhibitor as a marker highly expressed in the metastatic component of TNBC by analyzing a panel of 350 murine and 500 human secreted proteins. In the study conducted by Kozin *et al*. [25], it was demonstrated that SLPI is significantly more expressed in the TNBC 4 T1 cells, displaying a highly metastatic phenotype and capable of spontaneously forming distant metastases when injected orthotopically, compared to non‐metastatic ones. SLPI secretion correlates with the presence of lung metastases in injected mice, and the use of a specific SLPI inhibitor (C74) was shown to reduce lung metastases by 50% along with a concurrent reduction in primary tumor mass. These data were supported by the finding that patients with TNBC showed poor metastasis‐free survival (HR = 1.87, log‐rank*P* = 0.019) [25].

**Fig. 5:**
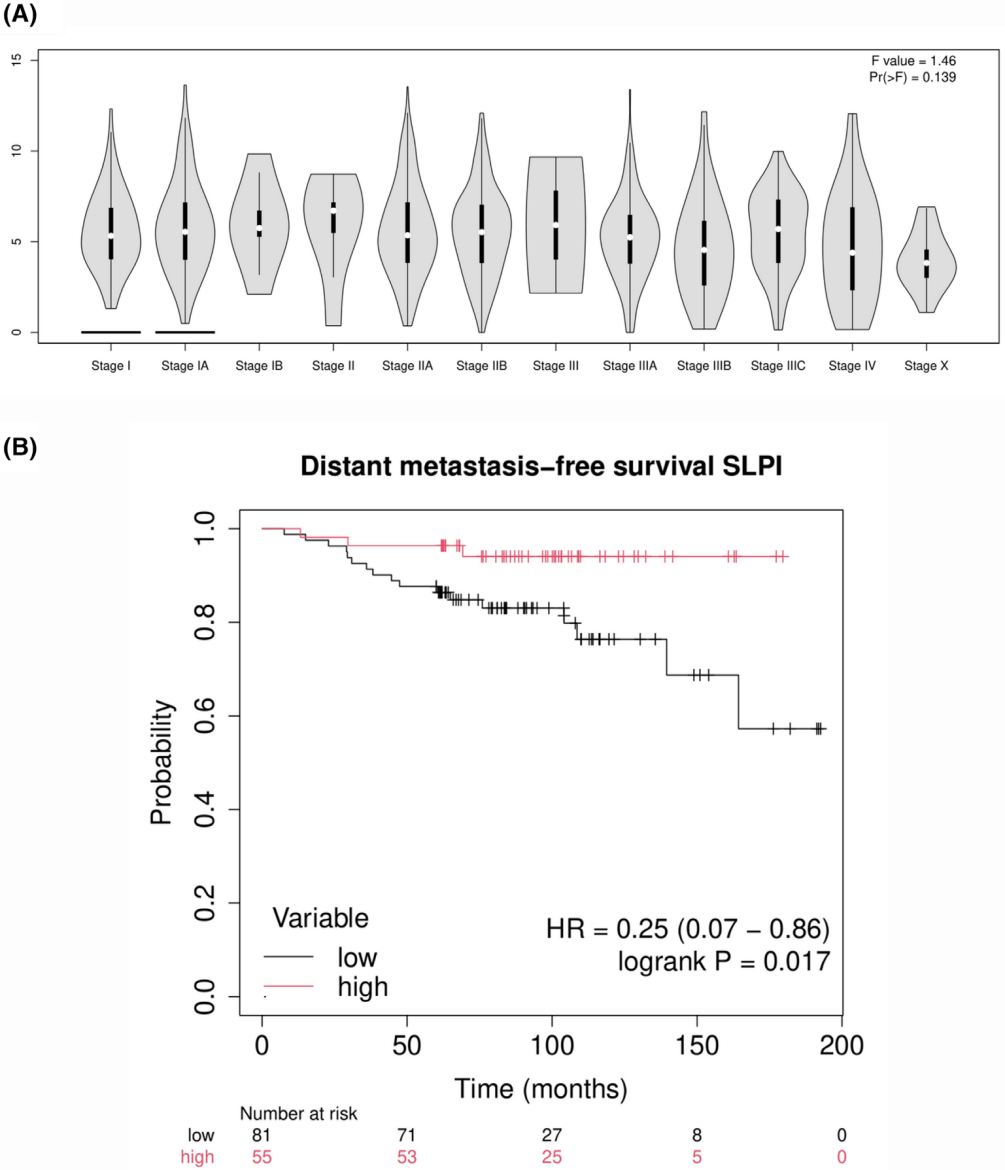
Investigating *SLPI* expression in primary tumors. (A) violin plots of *SLPI* expression in clinical stages of breast cancers (BrCas) with classification based on the tumor‐node‐metastasis (TNM) staging system. Clinical data were retrieved from The Cancer Genome Atlas (TGCA) and Genotype‐Tissue Expression (GTEx) databases. In the *Y* axis, the gene expression values are in log_2_(TPM + 1) (transcript per million). Stage I and IA present a horizontal line at the base of the violin plot, corresponding to samples where the *SLPI* expression was not detected (outliers). (B) the Kaplan–Meier plot of *SLPI* expression in high‐expressing (red line) and low‐expressing (black line) of BrCas from hormone‐positive tumors from the GSE12093 dataset (*P*‐value < 0.05).

To investigate whether potential confounding factors might have influenced our results, we examined *SLPI* expression across different clinical stages of BrCa (Fig. 5A) using GEPIA2 (http://gepia2.cancer‐pku.cn/, last accessed on 07 July 2025), based on TCGA/GTEx data. We applied a one‐way ANOVA test, which showed no significant variation in *SLPI* expression among tumor stages (*F* = 1.47, *P* = 0.139). Since the *P*‐value was not significant, we failed to reject the null hypothesis, indicating that intergroup differences were not greater than intragroup variation. Therefore, SLPI expression did not depend on tumor stage but may be influenced by basal‐like tumor subtype, including 80–86% of TNBCs (based on PAM50 classification).

To further validate our hypothesis, we used an independent dataset (GSE12093), which consists of 300 hormone receptor‐positive, lymph node‐negative BrCa from patients treated with tamoxifen, to generate a Kaplan–Meier curve using distant metastasis as the endpoint. As shown in Fig. 5B, the distant metastasis‐free survival analysis yielded HR = 0.25, indicating that high‐expressing tumors have a four‐fold lower risk of metastasis compared to low‐expressing tumors (log‐rank*P* = 0.017). Additionally, we performed a multivariate analysis using the web tool PrognoScan (https://dna00.bio.kyutech.ac.jp/PrognoScan/index.html, last accessed on 07 July 2025), which provided a Cox regression analysis of the same dataset, which revealed a statistically significant result (*P*‐value = 0.012).

**Fig. 6:**
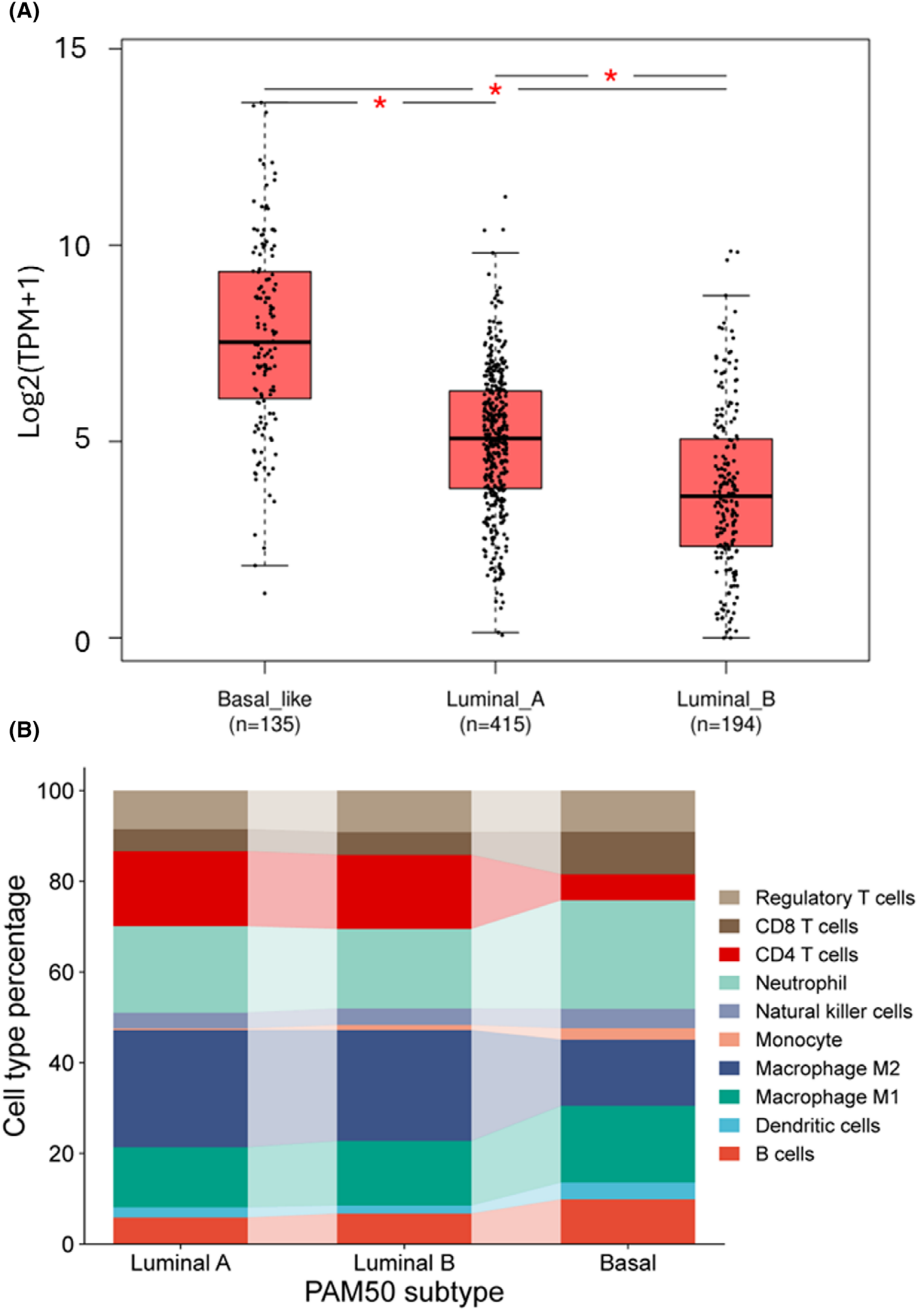
*SLPI* expression in breast cancer (BrCa) subtypes and immunome. (A) the boxplot shows *SLPI* gene expression across different BrCa prediction analyses of microarray 50 (PAM50) subtypes using the The Cancer Genome Atlas (TCGA) BRCA database from the GEPIA2 web tool (http://gepia2.cancer‐pku.cn/, last accessed on 08/07/2025). The red asterisks indicate significant differences between the compared pairs (*P*‐value < 0.05). Differential expression was assessed by one‐way ANOVA using disease state (Tumor vs. Normal) as the grouping variable. The horizontal line at the top and at the bottom of the boxes represents the maximum and minimum values of the category (whiskers) in −log_2_(TPM + 1) (transcript per million). (B) a stacked bar plot represents the percentage values of the different immune system components, calculated through deconvolution of RNA‐seq data from the TCGA BRCA database, obtained from The Cancer Immunome Atlas (https://tcia.at/home, last accessed on 08/07/2025).

Although *SLPI* high expression is a negative prognostic factor in metastatic basal‐like BrCa, in hormone‐positive and non‐metastatic tumors, it represents a favorable factor, independently of tumor stage. We further analyzed SLPI expression in basal‐like tumors compared to luminal A and luminal B subtypes. As shown in Fig. 6A, the latter exhibited significantly lower *SLPI* levels, and obtained the respective immune profiles by leveraging The Cancer Immunome Atlas (https://tcia.at/home, last accessed on 08 July 2025), which contains TCGA data, enabling the deconvolution analysis of RNA sequencing data from primary tumor biopsies. We generated the stacked bar plot (Fig. 6B) illustrating the different proportions of immune cells across the three tumor subtypes, revealing a marked reduction of CD4^+^ lymphocytes in basal‐like tumors compared to luminal subtypes. This difference was found to be significant by ANOVA test (*F* statistic = 148.6851, *P*‐value = 1.1102e‐16). We performed a *post hoc* Tukey's HSD analysis to pairwise compare the ANOVA results and to determine whether significant differences existed. This analysis revealed a substantial and statistically significant difference between the basal phenotype and each of the luminal subtypes considered individually, based on the highest *Q* value (Basal *vs* LumA, *Q* value = 23.34, *P* < 0.01; Basal *vs* LumB, *Q* value = 18.25, *P* < 0.01; LumA *vs* LumB, *Q* value = 4.27, *P* = 0.01). In the literature, it has been reported that the administration of exogenous SLPI induces a blockade of CD4^+^ T‐cell proliferation [29], suggesting that in basal‐like tumors, where SLPI expression is higher, this may result in a lower presence of CD4^+^ T lymphocytes, consistent with the findings from our current analysis.

A reduction in CD4^+^ T lymphocytes may indicate an immunosuppressed tumor microenvironment or one that is not favorable to exert an effective antitumor immune response. Numerous studies have shown that, particularly in advanced tumors, tumor‐associated neutrophils promote tumor growth, angiogenesis, and metastasis formation, and are often associated with poor prognosis.

### The overlapping expression of TNBCs and MLLs: A closer look at shared features

**Fig. 7:**
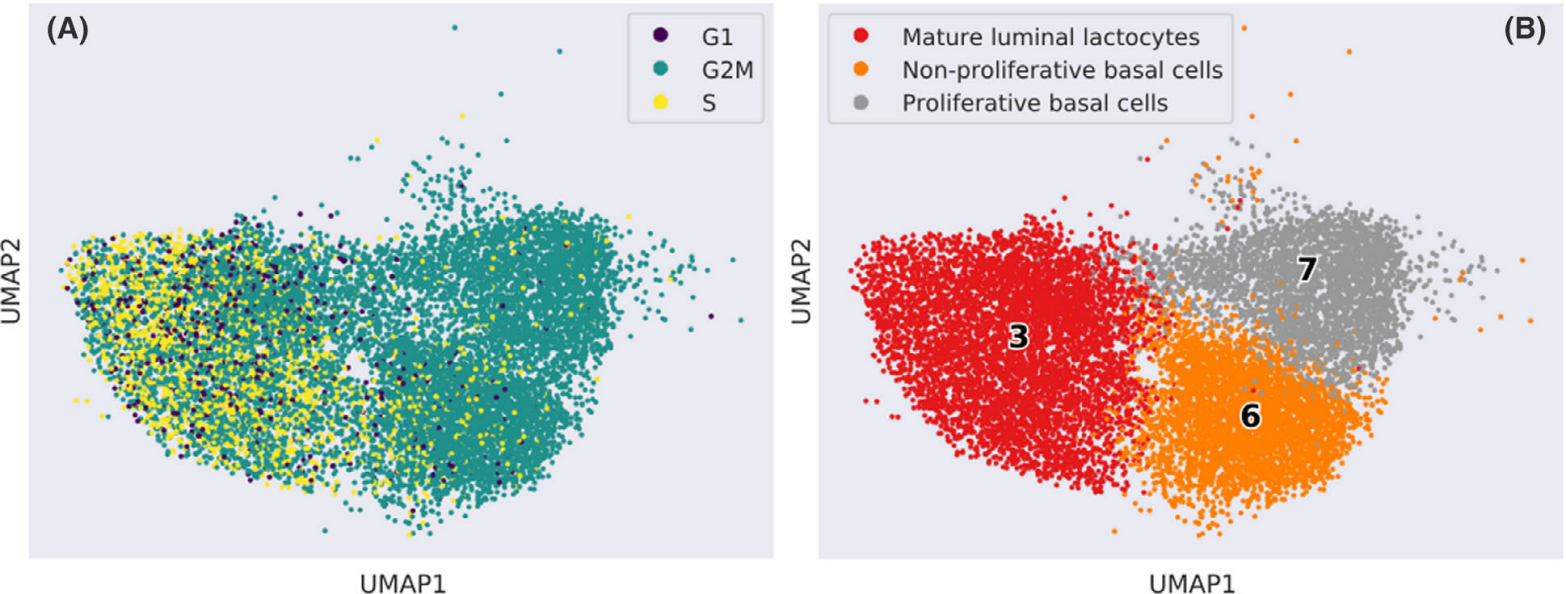
Cell cycle analysis. (A) the Uniform Manifold Approximation and Projection (UMAP) represents the cell cycle phase gene expression of considered cell types (mature luminal lactocytes, MLLs; proliferative basal cancer cells, PBCs; and nonproliferative basal cancer cells, NPBCs). (B) their UMAP representation where the clusters' numbers are reported as labels.

From our analysis, it emerged that the TNBC clusters (cl‐6 and cl‐7) displayed partially overlapping gene expression with MLLs (cl‐3) (Fig. 1B). Since these cells did not share the same lineage (TNBCs are basal, while MLLs are luminal cells), we deepened the analysis to highlight similarities and differences among these clusters. In the first step, we checked whether the analogies between TNBCs and MLLs were related to the cell cycle phase. Therefore, we ran a cell cycle enrichment analysis showing that TNBCs and MLLs were in different cell cycle phases, based on their expressed genes (Fig. 7A,B). We concluded that their similarity was evidently due to other factors not depending on cell cycle genes. Also, we inspected the cells derived from TNBCs that clustered among MLLs and *vice versa*, employing the copyKAT software [17]. This package enables us to infer the ploidy of a cell based on the CNV detected by RNA expression. Although derived from the milk of healthy donors, unexpectedly, almost 89% of the MLLs were assigned to aneuploids, as occurred in TNBCs, including cl‐6 with NPBCs = 96.1% and cl‐7 with PBCs = 98.7%. This phenomenon did not depend on a bias of the software and was observed only for MLLs. Indeed, in every other epithelial cluster, the aneuploidy composition was aligned to the expected value related to the sample nature (e.g., 2.5% of the LDCs, cl‐11; 82.5% of the LCs containing predominantly luminal cancer contributions, cl‐0 and cl‐16; 1.1% of the LPs, cl‐4; 30.5% of the MYO, cl‐8), as reported in Data S1. This result may be due to the CNV analysis depending on RNA expression data rather than DNA to infer ploidy.

To improve robustness and cross‐validate our findings, we also performed a CNV inferring analysis using the InferCNVpy software, a python‐based extension of BROAD Institute's InferCNV tool (inferCNV of the Trinity CTAT Project, https://github.com/broadinstitute/inferCNV, accessed on 10 July 2025). The comparison between the heatmap obtained using CopyKAT or InferCNVpy software confirmed similar inferred whole‐genome CNVs for the epithelial compartment (Fig. S7).

**Fig. 8:**
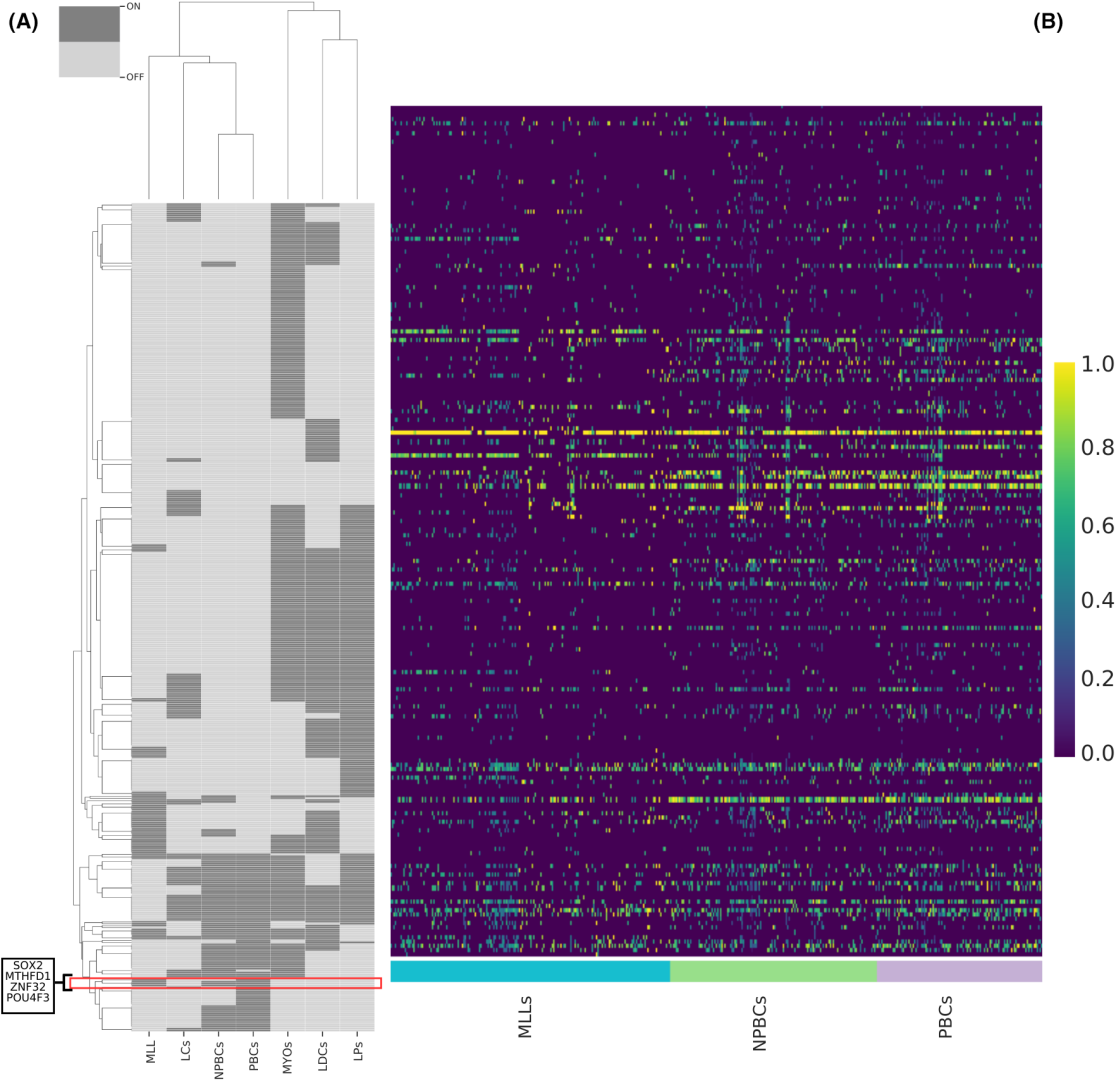
Gene regulatory network (GRN) comparison of mature luminal lactocytes (MLLs) to basal cancer cells. (A) the binarized heatmap of regulons derived from GRN analysis with pySCENIC. Dark gray blocks represent active regulons, while the light gray blocks are inactive; the red box highlights the common regulons active only in MLLs and basal cancer cells. (B) the heatmap of the 50 target genes under the transcriptional control of each considered regulon (MTHFD1, ZNF32, POU4F3, and SOX2) in MLLs, nonproliferative basal cancer cells (NPBC)s and proliferative basal cancer cells (PBCs) (total~200). Their increasing activation is indicated by the color ranging from purple (low) to yellow (high) in the bar scale.

Since TNBCs and MLLs have a similar pattern of gene expression, we pursued whether the two cell types shared common transcriptional program signatures. We employed the pySCENIC tool [16] to create GRN, highlighting the active “regulons” (Fig. 8A). We considered only transcripts coding TFs that were significantly altered in both scRNA differential expression analysis (DEA) and pseudo‐bulk DEA, setting log_2_FC (Fold Change) = ± 2 and FDR < 0.05 as thresholds, as reported in Data S2. We identified MTHFD1, ZNF32, POU4F3, and SOX2 as exclusive regulons, commonly active in mature lactocytes and cancerous basal cells, but inactive in the other cell types. The regulators MTHFD1, ZNF32, POU4F3, and SOX2 modulate numerous target genes. We selected a group of 50 target genes for each regulator, focusing on the most relevant ones, for a total of approximately 200 targets (some shared among different regulons, though not across all four). These targets were analyzed in MLLs, NPBCs, and PBCs (corresponding to cl‐3, cl‐6, cl‐7, clusters respectively), showing similar activation profiles, as shown in Fig. 8B. This homogeneous trend further confirms the efficacy of the GRN analysis.

**Fig. 9:**
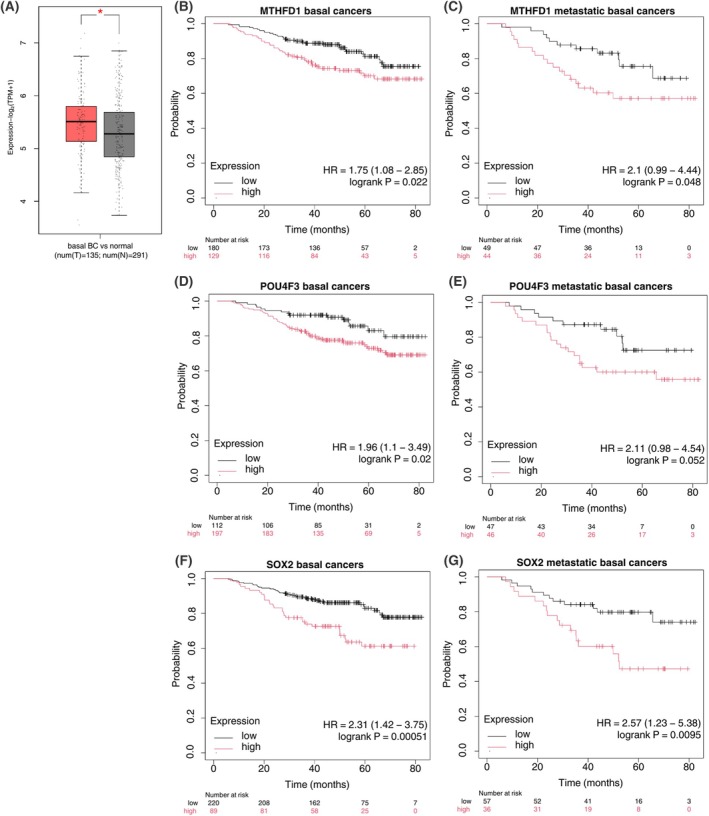
Overall survival (OS) plots and expression of key regulon of transcription factors (TFs). (A) the boxplot depicts the gene expression of MTHFD1 in basal breast cancer (BrCa) compared to normal tissue. Differential expression was assessed by one‐way ANOVA using disease state (Tumor vs. Normal) as the grouping variable. The horizontal line at the top and at the bottom of the boxes represents the maximum and minimum values of the category (whiskers) in –log_2_(TPM + 1) (transcript per million). The red asterisks indicate significant differences between the compared pairs (*P*‐value < 0.05). The Kaplan‐Meier plots depict the OS related to *MTHFD1* expression in basal BrCa (B) and metastatic basal BrCa (C); OS related to *POU4F3* in basal BrCa (D) and metastatic basal BrCa (E); OS related to *SOX2* in basal BrCa (F) and metastatic basal BrCa (G). In the X axis, the time is in months, while in the Y axis, the probability of overall survival is in decimals (0–1).

Focusing on the key factors of the regulons, we carried out an OS and expression analysis by assessing clinical data derived from GTEx and TCGA projects, and our findings are reported in Fig. 9. MTHFD1 is an important player in cellular metabolism and cancer‐related processes. MTHFD1 is an enzyme involved in the interconversion of one‐carbon derivatives of tetrahydrofolate, which are substrates for methionine, thymidylate, and *de novo* purine synthesis [30]. On the one hand, single‐nucleotide polymorphisms (SNPs) in *MTHFD1* were associated with a high content of choline in milk [31]; on the other hand, SNPs in this gene proved an increased risk to develop BrCa in Georgian [32] and invasion/metastasis in Iranian women [33]. This enzyme is modulated in various cancer processes [34] and extends its role beyond metabolic function, participating in DNA damage response and repair, essential for cancer cell survival. Using clinical data derived from GTEx and TCGA projects, MTHFD1 resulted upregulated in breast basal cancers compared to normal tissue (Fig. 9A). Moreover, MTHFD1 was found to be a negative prognostic marker in breast nonmetastatic (HR = 1.75, logrank*P* = 0.022, Fig. 9B), and in metastatic basal cancers (HR = 2.1, logrank*P* = 0.048, Fig. 9C), underlying its involvement in TNBCs. Targeting MTHFD1 and related enzymes has already been proposed as a therapeutic strategy to disrupt nucleotide synthesis and induce replication stress, leading to cancer cell death in prostate cancer [35]; this approach might make sense also to fight BrCa.

The other regulon under the control of POU4F3 has been evidenced as a master regulator of the survival of auditory sensory hair cells; indeed, when mutated, it led to complete hearing loss [36]. However, POU4F3's role in cancer is still under debate. A comprehensive study reported that POU4F3 facilitated the metastasis detection in sentinel lymph nodes of Merkel cell carcinoma [37]. On the contrary, another research group described POU4F3 as a tumor suppressor in lung adenocarcinoma [38]. Interestingly, the methylation status of *POU4F3* represents a diagnostic epigenetic biomarker test for risk assessment of high‐grade cervical intraepithelial neoplasia and cervical cancer in hrHPV‐positive women [39]. In the context of breast gland, there is no clear evidence of involvement in cancer, but a study conducted in Pakistan smallholder farming aimed to improve productivity levels through genomic selection of native cattle breeds, associated *POU4F3* with milk production, immunity, and adaptation traits in improving the low productivity levels [40]. We investigated the clinical value of this TF on the OS of basal BrCa. Although we did not observe differences between normal and primary tumors, a negative role of *POU4F3* in the survival of patients is significantly appreciable with basal cancers (HR = 1.96, logrank*P* = 0.02, Fig. 9D) and metastatic cancers (HR = 2.11, logrank*P* = 0.052, Fig. 9E), with a clear trend in the latter, even if not significant.

As we found analogies between TNBCs and MLLs, the physiological function of ZNF32 could be hijacked and exploited by tumor cells. Concerning the ZNF32 regulon, previously published articles reported discordant findings, suggesting both anti‐ and protumoral behaviors [41, 42]. In BrCa, this protein confers a stem‐like phenotype driving changes in focal adhesions and ECM–receptor interactions [43] and *ZNF32* upregulation promoted transcriptional changes in genes belonging to critical pathways associated with cancer proliferation, adhesion, and migration [43]. It exerts a protective role against Akt/mTOR inhibitor‐induced autophagy, as demonstrated by silencing *ZNF32* with siRNA in Xenograft BrCa models [44].

Concerning the last TF emerging from our analysis, SOX2 plays a role in several diseases. In physiological conditions, SOX2 drives embryo development, regulating stemness‐related features, and is crucial as demonstrated using SOX2^−/−^ mice embryos, which die right after implantation [45]. The impact of SOX2 in tumor development is strictly type‐related, and its overexpression was linked to cell proliferation, epithelial‐to‐mesenchymal transition, cell death escaping, and drug resistance [46]. In BrCa, *SOX2* is strongly expressed, especially in TNBC, in which it is considered a negative prognostic factor correlated with poor survival [47]. However, we did not find significantly different levels of *SOX2*, comparing breast basal cancers and normal tissues. We validated the prognostic value of SOX2 by assessing clinical TGCA and GTEx data, in which SOX2 has predictive value for OS in both breast basal cancers (HR = 2.1, logrank*P* < 0.01, Fig. 9F) and metastatic breast basal cancers (HR = 2.57, logrank*P* < 0.01, Fig. 9G).

The coactivation of these four factors suggests a conserved mechanism that integrates metabolic and transcriptional networks to support both normal cellular functions and the aggressive behavior of cancer cells. Our results are consistent with other scRNA‐seq studies carried out on breast glands. Hu and colleagues suggested that BRCA1‐mutated ER^−^ basal‐like BrCas could derive from LPs after triggering basal‐like transformation [48]. Another study demonstrated an enrichment of MLLs in BRCA1/p53‐mutated BrCa‐bearing mice [49]. Finally, Molyneux and colleagues came to the same conclusions, deleting BRCA1 in murine mammary LPs, observing basal cancers resembling the sporadic ones [50].

Further information, concerning the relationship between these regulons and cancer (Data S3), derived from an enrichment analysis carried out using the gseapy package with the KEGG human gene lists [51]. Using the top 50 target genes modulated by each regulon as input, we found, among the enriched pathways, immunological responses such as IL‐17 signaling, viral protein interaction with cytokine and cytokine receptor, cytokine–cytokine receptor interaction, antigen processing and presentation, and cancer pathways, such as Wnt and p53 signaling and transcriptional misregulation (Fig. S8a–d). The unique common pathway is the Herpes simplex virus 1 infection, with a combined score of 168, odds ratio of 6.34, and FDR < 0.05 (Data S4). Using the same lists, we performed a network analysis by String (https://string‐db.org/, accessed 25 March 2025) indicating the downstream functional targets of the regulons and the interacting proteins, commonly modulated both in MLLs and TNBCs (Fig. S9a–e).

To confirm our findings obtained by regulons analysis, we analyzed the commonly upregulated markers between MLLs and TNBCs based on ploidy information, inferring most of MLLs as aneuploid. We hypothesized that this misleading prediction is due to a similar expression pattern displayed by these cells. We extracted the MLL profiles tagged as “aneuploid” and explored their markers using the Wilcoxon rank‐sum test. We filtered significantly upregulated genes from TNBCs and MLLs (FDR < 0.05) finding 876 significantly shared top markers (Data S5), which are automatically ranked based on the “score,” representing the test statistic (*U*‐value) from the Wilcoxon rank‐sum test [52]. The average between the *U*‐value of TNBCs and MLLs was obtained to extract crucial genes from both conditions. The top 100 genes were employed as an input list for pathway analysis, which revealed a significant enrichment (FDR < 0.05) of different KEGG pathways (Table 1). Among them, the ribosome pathway was prominent with a score of 37.3.

**Table 1:** Gene set enrichment analysis of KEGG pathways. The table comprises enrichment scores and annotations of KEGG pathways, including the gene set code, the description of the pathway, the size of the whole gene list for each pathway, the expected hits for the given pathway, the ratio score (observed hits over expected hits), and the associated *P*‐value and false discovery rate (FDR) for each enrichment analysis.

Gene set	Description	Size	Expect	Ratio	*P*	FDR
hsa03010	Ribosome	167	1.7967	37.29	2.11E‐99	7.42E‐97
hsa05171	Coronavirus disease	232	2.4961	26.842	8.23E‐88	1.45E‐85
hsa00190	Oxidative phosphorylation	134	1.4417	7.6299	1.70E‐7	1.97E‐5
hsa05415	Diabetic cardiomyopathy	203	2.1841	5.9522	2.24E‐7	1.97E‐5
hsa05012	Parkinson disease	266	2.8619	4.8919	8.17E‐7	5.75E‐5
hsa05208	Chemical carcinogenesis	223	2.3992	5.0016	4.26E‐6	2.50E‐4
hsa04714	Thermogenesis	232	2.4961	4.8076	6.41E‐6	3.22E‐4
hsa05016	Huntington disease	306	3.2922	3.9487	2.16E‐5	9.49E‐4
hsa05020	Prion disease	272	2.9264	4.1006	3.18E‐5	1.24E‐3
hsa04260	Cardiac muscle contraction	87	0.93602	7.4784	3.86E‐5	1.36E‐3

The activation of SOX2 regulon in these cells appears to be tightly integrated with their metabolic state. Both ribosomal and oxidative phosphorylation pathways suggest a high demand for energy. Given that SOX2 established a role in cellular metabolism and it is linked to ATP production [53], these findings create a cohesive picture: SOX2 may be actively driving metabolic processes necessary for elevated levels of protein synthesis and energy generation within these cells. The Leucine‐Rich Repeat Containing G Protein‐Coupled Receptor 4 (LGR4)‐mediated signaling drives Wnt signaling that regulates *SOX2*. Wang and colleagues described a correlation between SOX2 and LGR4, a Wnt receptor and positive regulator of *SOX2* [54]. The overexpression of *SOX2* in LGR4^−/−^ mammary cells rescues their impaired self‐renewal capabilities *in vitro* (colony formation) and *in vivo* (ductal outgrowth). These findings suggest that SOX2 is a critical downstream effector of LGR4/Wnt signaling for mammary stem cell maintenance and functions [54]. In agreement with this evidence, Wnt signaling was activated also in TNBCs and MLLs as demonstrated by our enrichment analysis of the top 50 targets of MTHFD1 regulon (Fig. S9d).

Since both TNBCs and MLLs showed the activation of the SOX2 regulon, this suggests a common signaling pathway associated with stem‐like features, which conversely is inactive in the other cell types. Indeed, SOX2, one of the four TFs exclusively expressed in both TNBC and lactocytes and strongly associated with mammary gland self‐renewal, plays a crucial role during pregnancy and lactation. Its expression levels significantly influence maternal and neonatal phenotypic traits, including birthweight, changes in breast size, and gestational age at delivery [55]. CLDN4 is also expressed in both TNBCs and lactocytes; in addition to being a marker of mammary stem cells, it is upregulated during lactation [56]. However, it remains challenging to determine whether this overlap is due to shared developmental mechanisms reminiscent of mammary gland maturation or instead linked to a “milk‐associated” tumor phenotype. Indeed, several genes associated with milk production are also involved in fatty acid and triglyceride synthesis (Fig. S10). For example, diacylglycerol acyltransferase‐1 (DGAT1) and glycerol‐3‐phosphate acyltransferase 4 (GPAT4) catalyze reactions in triglyceride synthesis, which are essential for generating nutrient‐rich milk [57]. Other canonical “milk‐associated” genes include *FASN*, which catalyzes the *de novo* synthesis of fatty acids, *FOLR1*, essential for lactose production, and *B4GALT1*, encoding β‐1,4‐galactosyltransferase, an enzyme directly involved in lactose synthesis [58] that may reflect a reactivation of lactation‐related molecular programs. In addition, ornithine decarboxylase 1 (ODC1), expressed in both TNBCs and MLLs, is an enzyme critical for polyamine biosynthesis and a key regulator of protein synthesis and lactogenesis. Its transcription is stimulated by high nutritional levels and is significantly upregulated during the mammary gland expansion associated with lactation [59, 60].

To further investigate this dual aspect, involving either developmental pathways of the mammary gland or a milk‐associated cancerous phenotype, we analyzed the overlap between lactocytes and basal tumor cells using data from the *Mammary Gland Development Atlas* (https://github.com/MarioniLab/MammaryGland, accessed on 12 July 2025), which provides a detailed reconstruction of murine mammary gland development across the animal's entire lifespan. After downloading and preprocessing the dataset, including quality control steps, we proceeded with reference mapping, a bioinformatic approach for scRNA‐seq data that allows “query cells” (in our case, basal tumor cells) to be projected onto a reference dataset (murine mammary gland cells). This method integrates the embeddings of the reference and query datasets and aligns them using UMAP as a dimensionality reduction model. The query cells were then annotated and aligned to the reference via a K‐nearest neighbor classifier. As shown in Fig. S11a,b, the results of this analysis indicate that the majority of the basal tumor cells are projected onto UMAP coordinates corresponding to cluster C20 in the reference. According to the authors of the original dataset (https://doi.org/10.5281/zenodo.15631326, accessed on 12 July 2025), this cluster represents a rare cell population with intermediate features between cluster C8 (Differentiated Alveolar Cells, i.e., lactocytes) and cluster C13 (basal cells) of their study. These findings suggest that the cancerous basal cells in our study overlapped with lactocytes and exhibited a transcriptomic landscape resembling that of mature lactocytes. This is supported by the projection of our cells using the mammary gland reference dataset. Finally, a small subset of basal tumor cells was projected near cluster C4 in the UMAP (Hormone Sensing Differentiated Cells). However, the clustering algorithm labeled their gene expression profile as more similar to cluster C12, which corresponds to *bona fide* basal cells, consistent with the basal nature of our samples.

This last analysis suggests a predominantly milk‐associated profile, although these findings should be validated in human samples, which are currently unavailable.

### TNBCs and MLLs divergent expression: Absence of MALAT1 and NEAT1 in MLLs

**Fig. 10:**
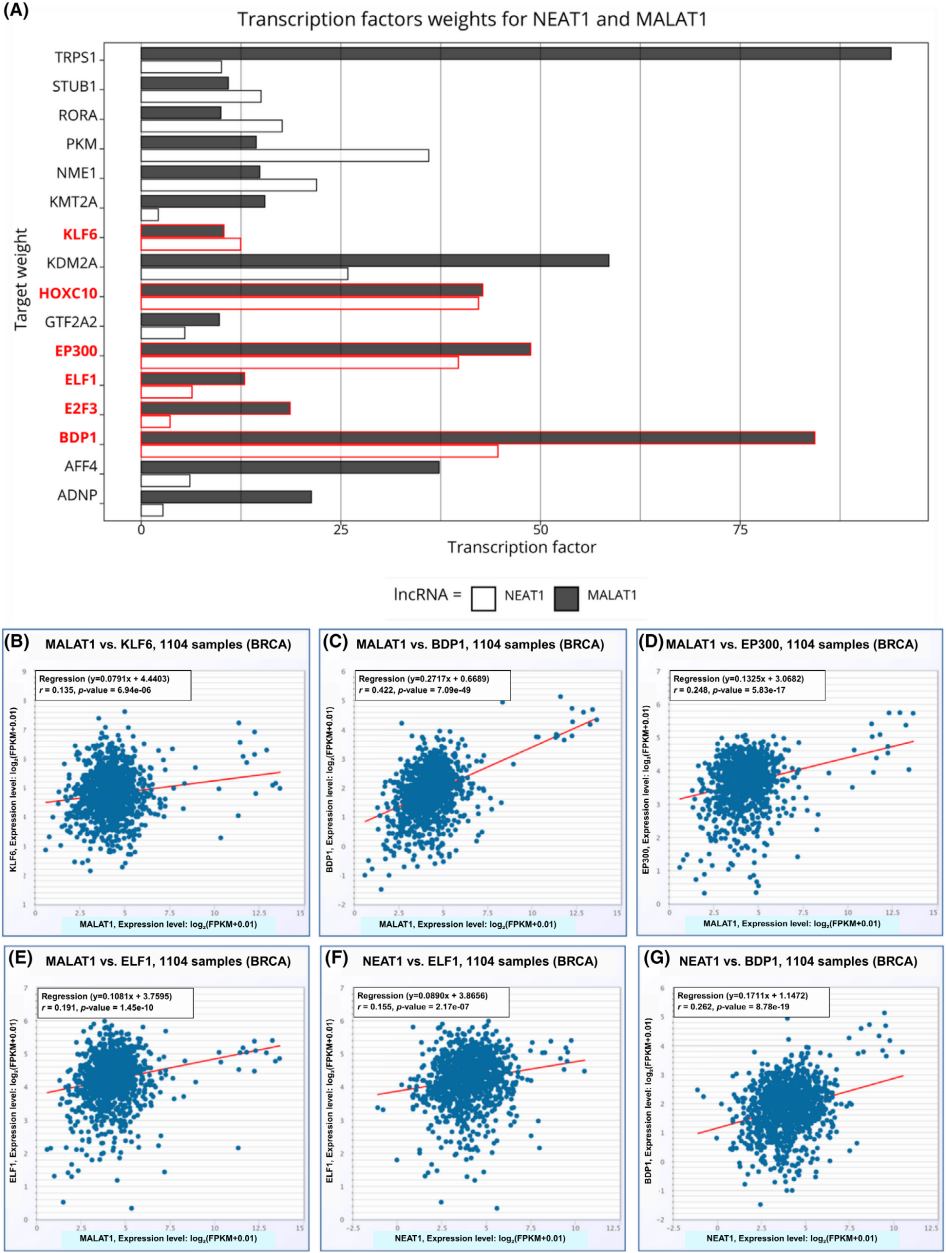
MALAT1, NEAT1 and co‐expression of transcription factors (TFs). (A) the bar plot of NEAT1 and MALAT1 interactions with all predicted TFs. The *X* axis represents the weights (strength) of the TF–target interaction, while in *Y*, the TFs are plotted. The dark gray bars represent MALAT1 values, while the white bars represent the NEAT1; in red, the 6 TFs left from area under the curve (AUC) analysis were highlighted. In (B–E), the dotplots portray the correlation of MALAT1 and TFs mRNA expression in The Cancer Genome Atlas (TCGA) clinical data (KLF6, BDP1, EP300, and ELF1 respectively). Finally, in F and G, the correlation of NEAT1 and ELF1 (F) and BDP1 (G) mRNA in TCGA data.

After investigating the similarities between TNBCs and MLLs, we focused on their divergences. Surprisingly, the long noncoding RNAs (lncRNAs) MALAT1 and NEAT1 were the most downregulated transcripts in MLLs using the Wilcoxon rank‐sum test. We focused only on the downregulated genes because those upregulated were predominantly associated with milk production in MLLs. This evidence is intriguing if we think that every other cell type considered in our analysis expresses these two lncRNAs at high levels. MALAT1 is one of the most enriched transcripts, owing to its steady and elevated expression among every human tissue [61], so much so that artifacts often occur during the amplification phase of sequencing [62]. For this reason, in most of the scRNA‐seq analyses, the MALAT1 expression is not taken into account and is considered biologically meaningless. In our case, we detected the absence of MALAT1 and NEAT1 that could represent a crucial insight in discerning the physiological and pathological functions of the breast gland. We investigated this phenomenon, first checking whether the lack of expressed lncRNAs was confirmed by regulon analysis. Data reported in the Data S6, which contain the predicted relationships between TFs and their potential target genes, are the output of the first step of the GRN analysis. We extracted all the predicted TFs having both MALAT1 and NEAT1 as targets. From these two lists, we considered the common with a median weight (which measures the strength of TF–target interaction) of at least 10, obtaining 16 TFs (Fig. 10A). Some of these TFs were ruled out in the successive steps of the analysis. Indeed, the adjacencies predicted values of the interactions, while the definitive regulons obtained from the area under the curve (AUC) analysis were those for which the inference resulted relevant. The scores (weights) were calculated based on the expression levels of the target genes in the regulon, and subsequently, TFs that were not active or predictive in the analysis were removed.

Therefore, we checked which of the 16 TFs were inferred as important regulons in the second step using pySCENIC, obtaining only six TFs contained in the final AUCell output (Data S6). Interestingly, KLF6, HOXC10, EP300, E2F3, BDP1, and ELF1 were all inactive in the MLLs, while at least one of them was active in the other epithelial or MYO cells, confirming that MALAT1 and NEAT1 null expression in MLLs is biologically meaningful and not derived from technical artifacts. To validate our results, we employed the GEPIA2 tool [24] to compare the expression of the two lncRNAs to that of each TF, using both TGCA and GTEx data.

**Fig. 11:**
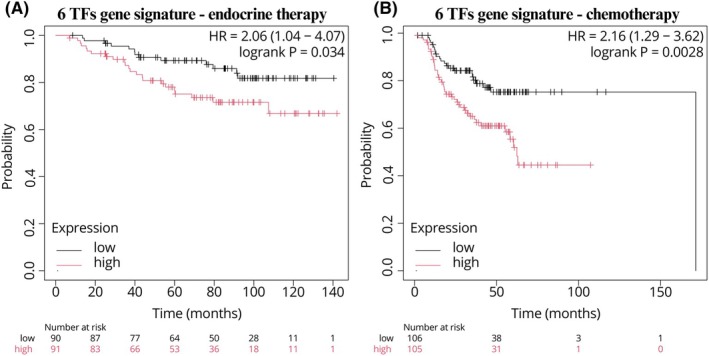
Relapse‐free survival (RFS) of the signature of the 6 transcription factors (TFs). The signatures of EP300, ELF1, KLF6, E2F3, BDP1, and HOXC10 were used to study the RFS of endocrine‐treated breast cancer (BrCa) patients (A), and chemo‐treated BrCa patients (B). In the *X* axis, the time in months, while in the *Y* axis, the probability of relapse‐free survival in decimals (0–1).

As shown in Fig. 10B–G, in TCGA clinical data extracted from starBase v2.0 webtool (https://rnasysu.com/encori/, accessed on 11 February 2025) [63], we found a significant positive correlation (*r*) between the expression of MALAT1 and four examined TFs (BDP1, ELF1, EP300, and KLF6) and the expression of NEAT1 and two TFs (BDP1 and ELF1). We hypothesize that MALAT1 and NEAT1 could represent a key in the switch from physiological to pathological cell growth of the breast gland. Both NEAT1 and MALAT1 have been extensively investigated as oncogenes in cancer, especially in BrCa [64, 65, 66]. Given the huge literature supporting this evidence, we employed clinical data to verify whether the six TFs, which strongly target and transcribe the two lncRNAs, could serve as gene signatures in BrCa outcome prediction. Our analysis revealed that the signature of these TFs could significantly predict the relapse‐free survival (RFS) of BrCa patients treated with endocrine therapy (HR = 2.06, logrank*P* = 0.034) (Fig. 11A) or chemotherapy (HR = 2.16, logrank*P* < 0.01) (Fig. 11B).

Using another scRNA‐seq dataset from the single‐cell portal of Broad Institute (https://singlecell.broadinstitute.org/single_cell, last accessed on 3 March 2025) [67], both MALAT1 and NEAT1 appeared downregulated in milk‐producing cells (indicated as luminal cluster 2, LC2, and highly expressing genes associated with milk synthesis) (Fig. S12a–d). This analysis conducted using another human dataset confirmed the lack of NEAT1 and MALAT1 in MLLs.

With the aim to further validate our results, we used a murine dataset, obtained using the RShinyapp named “Mammary Gland Development” from Marioni's laboratory (https://marionilab.cruk.cam.ac.uk/mammaryGland/, accessed on 10 February 2025). This analysis showed that MALAT1 is also downregulated in murine milk‐producing cells (Fig. S12e,f, named C8 representing differentiated alveolar cells), confirming our findings, and revealing a similar gene expression pattern constitutively and evolutionarily conserved in human and murine cells.

Despite the key involvement of MALAT1 and NEAT1 in cancer development and progression, it is hard to target them in therapeutic approaches since these lncRNAs are highly expressed in normal human tissues, which could be affected along with the cancerous cells. Furthermore, MALAT1 and NEAT1 are primarily localized in the nucleus, where RNA‐targeting drugs have limited access compared with cytoplasmic targets. Delivering antisense oligonucleotides, small interfering RNAs, or CRISPR/Cas 9‐based therapies represents strategies to affect specific tissues but present obstacles depending on the density and heterogeneity of the tumor microenvironment, making drug access difficult, and on the degradation by RNA nucleases in the bloodstream, requiring chemical modifications, which may alter efficacy or safety.

Our results potentially shed light on alternative approaches to tackle the relapse‐prone BrCa by targeting TFs. For example, EP300 bromodomain inhibitors, such as CZL‐046 [68] and the most recent OPN‐6602 currently undergoing a phase 1 clinical trial for multiple myeloma (NCT06433947) [69], should be investigated also for recurrent BrCa.

## Conclusion

Our analysis provides novel insights into the complex cellular heterogeneity of the mammary gland. Using a scRNA‐seq approach, we characterized the cell types derived from samples obtained from normal and cancerous breast tissues, as well as from breastfeeding tissue. We assigned a prognostic value to SLPI in both metastatic and nonmetastatic basal BrCa. SLPI was highly expressed in basal‐like subtypes compared to luminal ones, and its expression was not related to clinical stage and appeared to have a potential impact on the tumor microenvironment and its immune composition. Furthermore, our analysis, on the one hand, emphasized the transcriptional overlap between TNBCs and MLLs, although it remains unclear whether this is due to shared developmental mechanisms of the mammary gland or to a “milk‐associated” tumor phenotype. Based on the analysis of murine data from Mammary Gland Development Atlas, we hypothesize that cancerous basal cells may more closely resemble mature lactocytes. In addition, they shared the activation of four key regulons, which suggests conserved molecular mechanisms in driving both normal mammary development and tumorigenesis. MTHFD1 and SOX2 impacted OS, while the role of POU4F3 and ZNF32 deserves further investigation. On the other hand, our study suggests that the exclusive absence of MALAT1 and NEAT1 in MLLs opens new ways to understand BrCa progression and lactation biology. The different expression of six TFs (EP300, ELF1, E2F3, BDP1, HOXC10, and KLF6) related to these two lncRNAs and their RFS signatures warrants further exploration to decipher their potential as diagnostic biomarkers or therapeutic targets, opening new drug‐repurposing approaches for BrCa.

## Author contributions

PA designed the study; PA performed the experiments; PA, NB, CMB, CF, and SV analyzed the data; CMB., CF, and NB drafted the paper; CMB. and CF revised the manuscript.

## Conflict of interest

The authors declare no conflict of interest.

## Supporting information


**Table S1.** List of the samples analyzed. The table contains the key information: the bioassay and the original study codes (from GEO or ArrayExpress), the type of tissue, and the number of single‐cell profiles used in the prequality check.

**Fig. S1.** Uniform manifold approximation and projection (UMAP) comparison of the plots before and after batch correction. Single‐cell RNA (scRNA) profiles from the three investigated datasets (GSE161529, GSE245601, and E‐MTAB‐9841) were processed using scanpy.external.pp.harmony for batch correction. (a) shows the UMAP visualization before integration, while (b) displays the UMAP after Harmony integration with parameters: *lambda* = 1, *theta* = 2, *sigma* = 0.1.

**Fig. S2.** Stromal and immune cells markers. Granulysin (*GNLY*), *CD8A*, and *CD3D* were expressed in T cells/NK cells; *CD79A*, *MS4A1* Membrane Spanning and Mast Cell Carboxypeptidase A (*CPA3*) in B cells/mast cells; *CD68*, Interferon Regulatory Factor 8 (*IRF8*), and Lysozyme (*LYZ*) in dendritic cells (DCs)/macrophages; decorin (*DCN*), collagen family member *COL3A1*, and *LUM*, an ECM protein in fibroblasts; Adhesion G protein‐coupled receptor L4 (*ADGRL4*) and Platelet And Endothelial Cell Adhesion Molecule 1 (*PECAM1*) in endothelial cells; ATP Binding Cassette Subfamily C Member 9 (*ABCC9*), Platelet‐Derived Growth Factor Receptor β (*PDGFRB*), and Regulator of G Protein Signaling 5 (*RGS5*) in pericytes.

**Fig. S3.** Epithelial and myoepithelial (MYO) cells markers. Cytokeratin 8 (*KRT8*), E‐cadherin (*CDH1*), and forkhead box protein A1 (*FOXA1*) were used as luminal cells markers; actin alpha 2 (*ACTA2*), myosin light chain kinase (*MYLK*), and *TP63* were expressed in MYO cells; cytokeratin 5, 14 and 17 (*KRT5*, *KRT14*, *KRT17*) in basal cells; lactoferrin (*LTF*), lactalbumin alpha (*LALBA*), and butyrophilin subfamily 1 member A1 (*BTN1A1*) for mature luminal lactocytes; prominin 1 (PROM1), SRY‐Box transcription factor 10 (SOX10), and *TLR2* for LPs; *MUC4*, protocadherin 9 (*PCDH9*), and glutamate receptor‐interacting protein 1 (*GRIP1*) were used for differentiating luminal cells.

**Fig. S4.** Cell cycle‐related Violin plots. Violin plots of proliferation‐related genes (*MKI67* in a, *TOP2A* in b, and *PCNA* in c), and G2/M score (d). In the *X* axis, the different cell types are displayed, while the *Y* axis represents the expression value for each gene (a–c) or the G2/M score (d).

**Fig. S5.** Uniform manifold approximation and projections (UMAPs) of proliferative basal cancer cells (PBCs), nonproliferative basal cancer cells (NPBCs), and myoepithelial (MYO) cells for MYO markers. This multipanel figure displays the normalized expression level of selected genes for MYO cell characterization. Each subpanel represents a different gene, with color intensity ranging from gray (low or no expression) to bright red (high expression), as indicated by the common color scale bar on the right of each plot.

**Fig. S6.** Differential gene expression of the top 30 markers upregulated in myoepithelial (MYO)‐like cancer cells. This heatmap displays the normalized expression levels of the top 30 genes identified as markers of aneuploid MYO‐like cancer cells. Each row represents a single cell from either healthy breast tissue or triple‐negative breast cancer (TNBC) patient tumors, as indicated by the blue (healthy_tissue) and light blue (tumor_TNBC) sidebar annotation on the *Y*‐axis. The *X*‐axis lists the top 30 differentially upregulated genes. The color intensity within the heatmap reflects gene expression levels, with darker shades (black/dark red) indicating lower expression and brighter shades (pink/yellow/white) indicating higher expression, as shown by the color scale bar.

**Fig. S7.** Copy number variation (CNV) heatmap of epithelial cells. The two heatmaps display inferred whole‐genome CNVs for individual epithelial cells using the CopyKAT (a) and InferCNVpy (b) software tools. Chromosomes are ordered sequentially along the X‐axis, and each row on the Y‐axis represents a single cell. In the heatmaps, color intensity reflects the genome copy number status, where blue indicates deletions and red indicates chromosomal amplifications. In a, cells grouped by the orange sidebar are computationally inferred as aneuploid (malignant), while those grouped by the green sidebar are inferred as diploid (nonmalignant/normal). In b, cells are grouped by the colored sidebar according to their annotation.

**Fig. S8.** Enrichment plots of common regulons. Bar plots of pathway enrichment analysis. The *X* axis represents the significance as –log_10_(*P*‐value), while in the *Y* axis, the different pathways are listed.

**Fig. S9.** Gene networks of top 50 targets of the four uniquely active regulon (SOX2, MTHFD1, ZNF32, and POU4F3). The nodes in the networks represent the input genes (top 50 targets of each regulon) and their first shell of 10 interactors based on scientific evidence. The thickness of the lines represents the strength of the evidence of interactions between two nodes, setting medium confidence as threshold. Made with https://string‐db.org/ accessed on 25 March 2025.

**Fig. S10.** Expression of genes shared by triple‐negative breast cancer cells (TNBCs) and mature luminal lactocytes (MLLs) and upregulated during lactation. Each panel shows the expression of the indicated gene in the two cell types.

**Fig. S11.** Reference mapping of query cells onto the mammary gland development atlas. (a) the uniform manifold approximation and projection (UMAP) of the reference dataset (mouse mammary gland development atlas) where cells are colored according to their annotated cluster. (b) the transcriptomic profiles of the query cells (nonproliferative basal cancer cells, NPBC; proliferative basal cancer cells, PBC) were mapped onto the UMAP space defined by the reference atlas using reference mapping (the scanpy ingest integrated tool). Reference cells are shown in light gray to provide contextual embedding, while query cells are highlighted in red.

**Fig. S12.** MALAT1 and NEAT1 validation. On the top panel, representing the Alexandria project data (https://singlecell.broadinstitute.org/single_cell?scpbr=the‐alexandria‐project), in a and c, the uniform manifold approximation and projections (UMAP) of MALAT1 and NEAT1 expression respectively; in b, the UMAP representation of single‐cell clusters annotated by cell types; in d, the cell labels corresponding to the cell types of the different clusters of b. The data and graphs were obtained using the BROAD institute Single Cell Portal (https://singlecell.broadinstitute.org/single_cell, last accessed on 03/03/2025). On the bottom panel, in e, the *t*‐distributed stochastic neighbor embedding (*t‐*SNE) representation of *Malat1* (ENSMUSG00000092341) expression in murine mammary tissue; in f, the corresponding boxplot where in the *X* axis, the different clusters are listed, while *Y* is the expression value. The black dotted circles highlight the clusters of interest representing the milk‐producing cells (differentiated alveolar cells or mature luminal lactocytes). Graphs in e and f have been obtained using the RShiny app from the Marioni Lab (https://marionilab.cruk.cam.ac.uk/mammaryGland/).

**Data S1.** Ploidy prediction results. This table contains the ploidy prediction results deriving from the copy number variation (CNV) analysis run with the copykat software. The table reports the absolute number of aneuploid cells, and the associated percentage compared to the total number of cells of the considered group. The results are reported considering both the different tissues and the different cell types.
**Data S2**. Significant altered regulons of pseudo‐bulk and single‐cell RNA (scRNA) differential expression analysis. This table reports the list of differentially expressed regulons according to both pseudo‐bulk and scRNA differential analysis using false discovery rate (FDR) < 0.05 and log_2_FoldChange (log_2_FC) < 2 or > 2 as significance threshold.
**Data S3**. Top 50 targets genes of mature luminal lactocytes (MLLs) and triple‐negative breast cancer cells (TNBCs) exclusively active regulons. This table contains the lists of the top 50 targets genes based on the gene regulatory network (GRN) prediction results of each of the considered regulon (ZNF32, SOX2, MTHFD1, and POU4F3).
**Data S4**. Herpes simplex virus 1 infection pathway enrichment results. The table reports the involved genes of the commonly enriched herpes simplex virus 1 infection pathway for each of the different considered regulon according to gseapy software analysis using the KEGG human gene lists.
**Data S5**. Common differentially expressed genes of aneuploid mature luminal lactocytes (MLLs) and triple‐negative breast cancer cells (TNBCs). This table contains information (gene names, scores, log_2_FoldChange (log_2_FC), and *p*‐values) of the commonly altered genes between aneuploid MLLs and TNBC cells.
**Data S6**. Gene Regulatory Network (GRN) results. This file reports the area under the curve (AUC) score of each regulon for each considered epithelial cell type. The values have been also reported as binarized (0 = inactive regulon, 1 = active regulon).

## Data Availability

All data needed to evaluate the conclusions in the paper are present in the paper and/or in the Supporting Information; raw data are freely available at the reported databases.
