# Supplementary material for: From lactation to malignancy: A comparison between healthy and cancerous breast gland at single‐cell resolution reveals new issues for tumorigenesis
Source: FEBS Lett. 2025 Sep 8;599(21):3124–49. doi: 10.1002/1873-3468.70162 (PMC12599593; doi:10.1002/1873-3468.70162)
Supplement: Supplementary file 1 — Table S1. List of the samples analyzed. The table contains the key information: the bioassay and the original study codes (from GEO or ArrayExpress), the type of tissue, and the number of single‐cell profiles used in the prequality check. [file FEB2-599-3124-s007.docx]

**Supplementary Table 1**. **List of the analyzed samples**. The table contains the key information: the bioassay and the original study codes (from GEO or ArrayExpress), the type of tissue, and the number of single-cell profiles used in the pre-quality check.

| SAMPLE | STUDY | TYPE | nCELLS |
| --- | --- | --- | --- |
| GSM7845558 | GSE161529 | tumor_TNBC | 3666 |
| GSM4909282 | GSE161529 | tumor_TNBC | 15870 |
| GSM4909283 | GSE161529 | tumor_TNBC | 1065 |
| GSM4909284 | GSE161529 | tumor_TNBC | 2015 |
| GSM4909289 | GSE161529 | tumor_HER2 | 4779 |
| GSM4909290 | GSE161529 | tumor_HER2 | 12288 |
| GSM4909291 | GSE161529 | tumor_HER2 | 4737 |
| GSM4909292 | GSE161529 | tumor_HER2 | 618 |
| GSM7845552 | GSE245601 | tumor_ERpos | 7463 |
| GSM7845554 | GSE245601 | tumor_ERpos | 5901 |
| GSM7845556 | GSE245601 | tumor_ERpos | 4820 |
| GSM7845558 | GSE245601 | tumor_ERpos | 1994 |
| ERR4903933 | E-MTAB-9841 | healthy_tissue | 9820 |
| ERR4903934 | E-MTAB-9841 | healthy_tissue | 11831 |
| ERR4903935 | E-MTAB-9841 | healthy_tissue | 15194 |
| ERR4903936 | E-MTAB-9841 | healthy_tissue | 10559 |
| ERR4903929 | E-MTAB-9841 | milk | 13865 |
| ERR4903930 | E-MTAB-9841 | milk | 3505 |
| ERR4903931 | E-MTAB-9841 | milk | 6916 |
| ERR4903932 | E-MTAB-9841 | milk | 6821 |
