# Supplementary figures and images for: From lactation to malignancy: A comparison between healthy and cancerous breast gland at single‐cell resolution reveals new issues for tumorigenesis
Source: FEBS Lett. 2025 Sep 8;599(21):3124–49. doi: 10.1002/1873-3468.70162 (PMC12599593; doi:10.1002/1873-3468.70162)

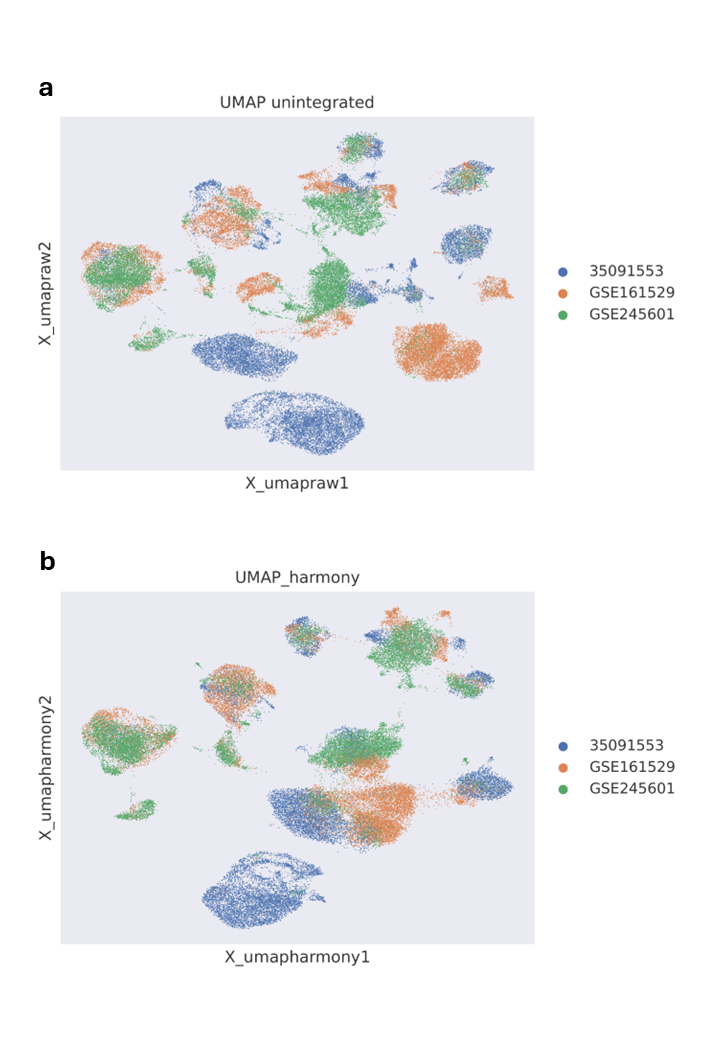

Supplement: Supplementary file 2 — Fig. S1. Uniform manifold approximation and projection (UMAP) comparison of the plots before and after batch correction. Single‐cell RNA (scRNA) profiles from the three investigated datasets (GSE161529, GSE245601, and E‐MTAB‐9841) were processed using scanpy.external.pp.harmony for batch correction. (a) shows the UMAP visualization before integration, while (b) displays the UMAP after Harmony integration with parameters: lambda = 1, theta = 2, sigma = 0.1. [file FEB2-599-3124-s003.tif]

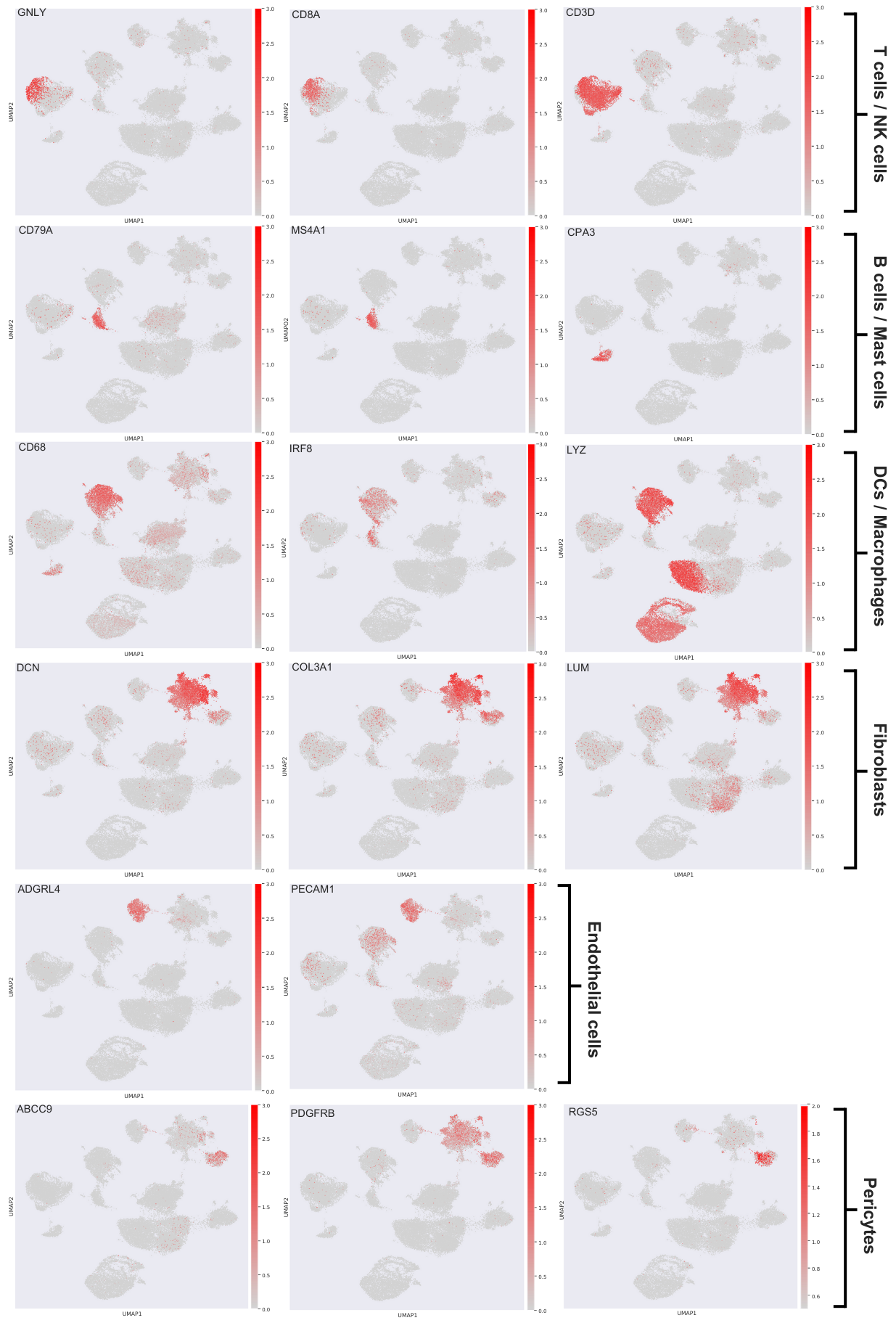

Supplement: Supplementary file 3 — Fig. S2. Stromal and immune cells markers. Granulysin (GNLY), CD8A, and CD3D were expressed in T cells/NK cells; CD79A, MS4A1 Membrane Spanning and Mast Cell Carboxypeptidase A (CPA3) in B cells/mast cells; CD68, Interferon Regulatory Factor 8 (IRF8), and Lysozyme (LYZ) in dendritic cells (DCs)/macrophages; decorin (DCN), collagen family member COL3A1, and LUM, an ECM protein in fibroblasts; Adhesion G protein‐coupled receptor L4 (ADGRL4) and Platelet And Endothelial Cell Adhesion Molecule 1 (PECAM1) in endothelial cells; ATP Binding Cassette Subfamily C Member 9 (ABCC9), Platelet‐Derived Growth Factor Receptor β (PDGFRB), and Regulator of G Protein Signaling 5 (RGS5) in pericytes. [file FEB2-599-3124-s004.tiff]

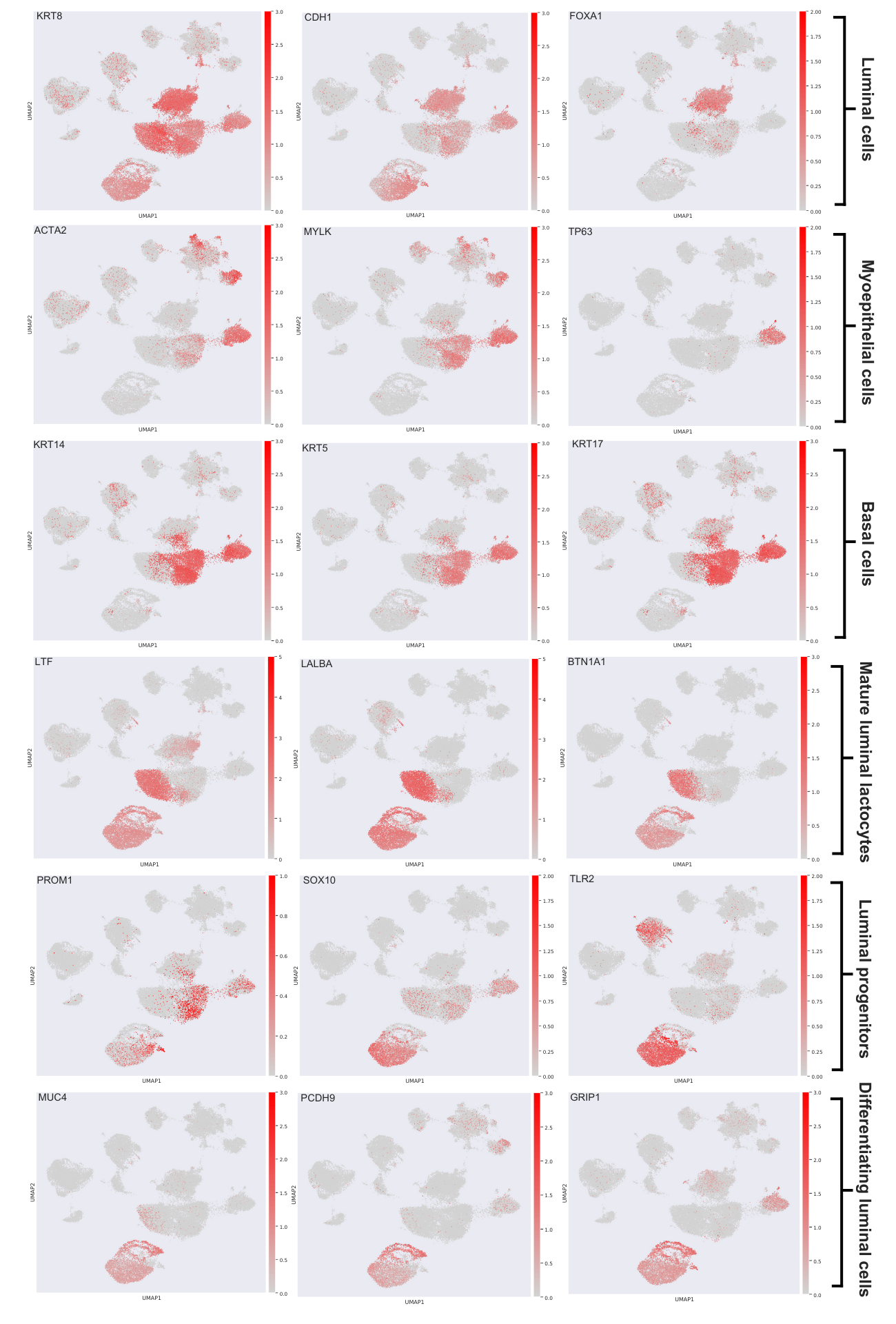

Supplement: Supplementary file 4 — Fig. S3. Epithelial and myoepithelial (MYO) cells markers. Cytokeratin 8 (KRT8), E‐cadherin (CDH1), and forkhead box protein A1 (FOXA1) were used as luminal cells markers; actin alpha 2 (ACTA2), myosin light chain kinase (MYLK), and TP63 were expressed in MYO cells; cytokeratin 5, 14 and 17 (KRT5, KRT14, KRT17) in basal cells; lactoferrin (LTF), lactalbumin alpha (LALBA), and butyrophilin subfamily 1 member A1 (BTN1A1) for mature luminal lactocytes; prominin 1 (PROM1), SRY‐Box transcription factor 10 (SOX10), and TLR2 for LPs; MUC4, protocadherin 9 (PCDH9), and glutamate receptor‐interacting protein 1 (GRIP1) were used for differentiating luminal cells. [file FEB2-599-3124-s013.tiff]

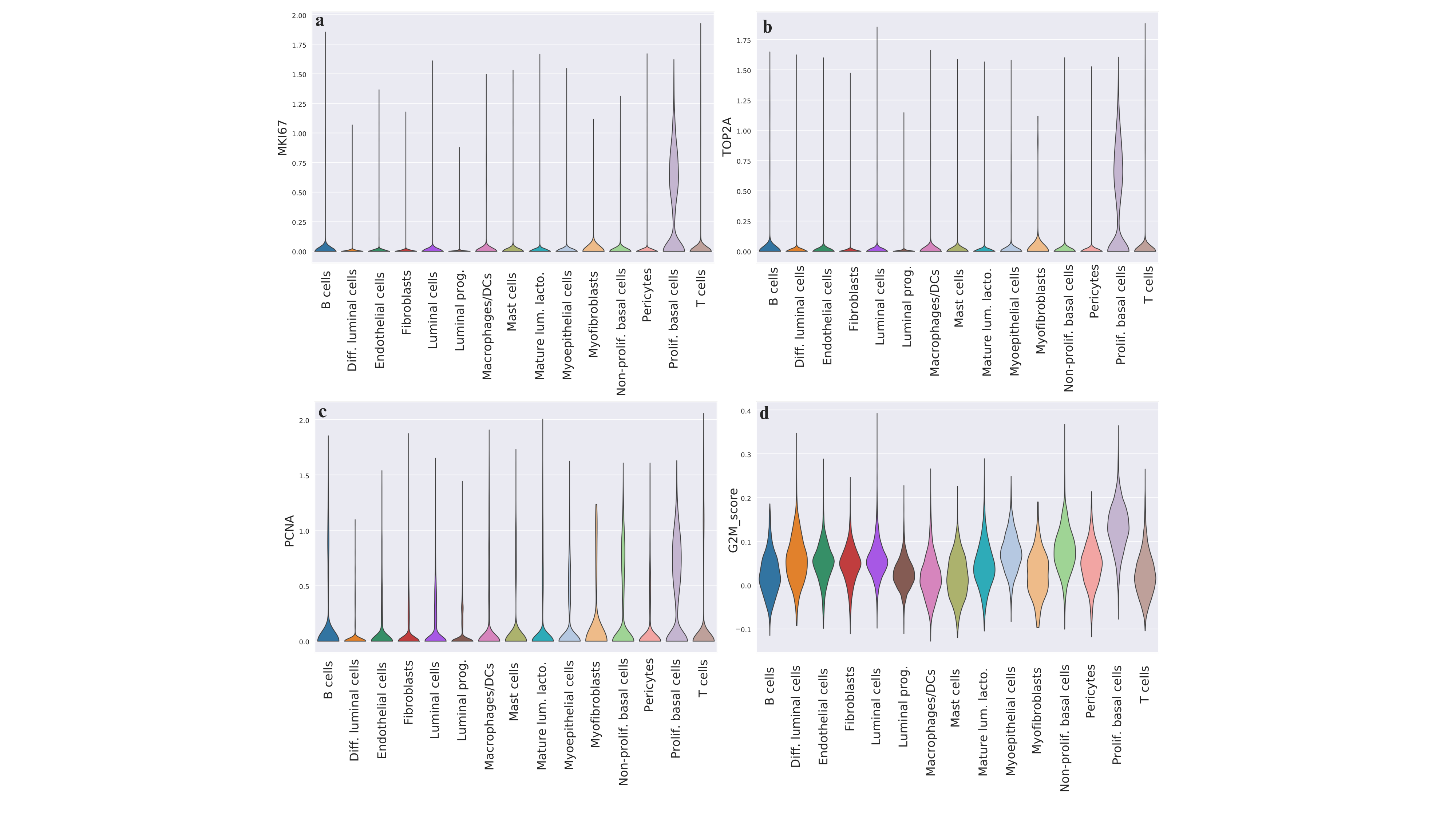

Supplement: Supplementary file 5 — Fig. S4. Cell cycle‐related Violin plots. Violin plots of proliferation‐related genes (MKI67 in a, TOP2A in b, and PCNA in c), and G2/M score (d). In the X axis, the different cell types are displayed, while the Y axis represents the expression value for each gene (a–c) or the G2/M score (d). [file FEB2-599-3124-s011.tiff]

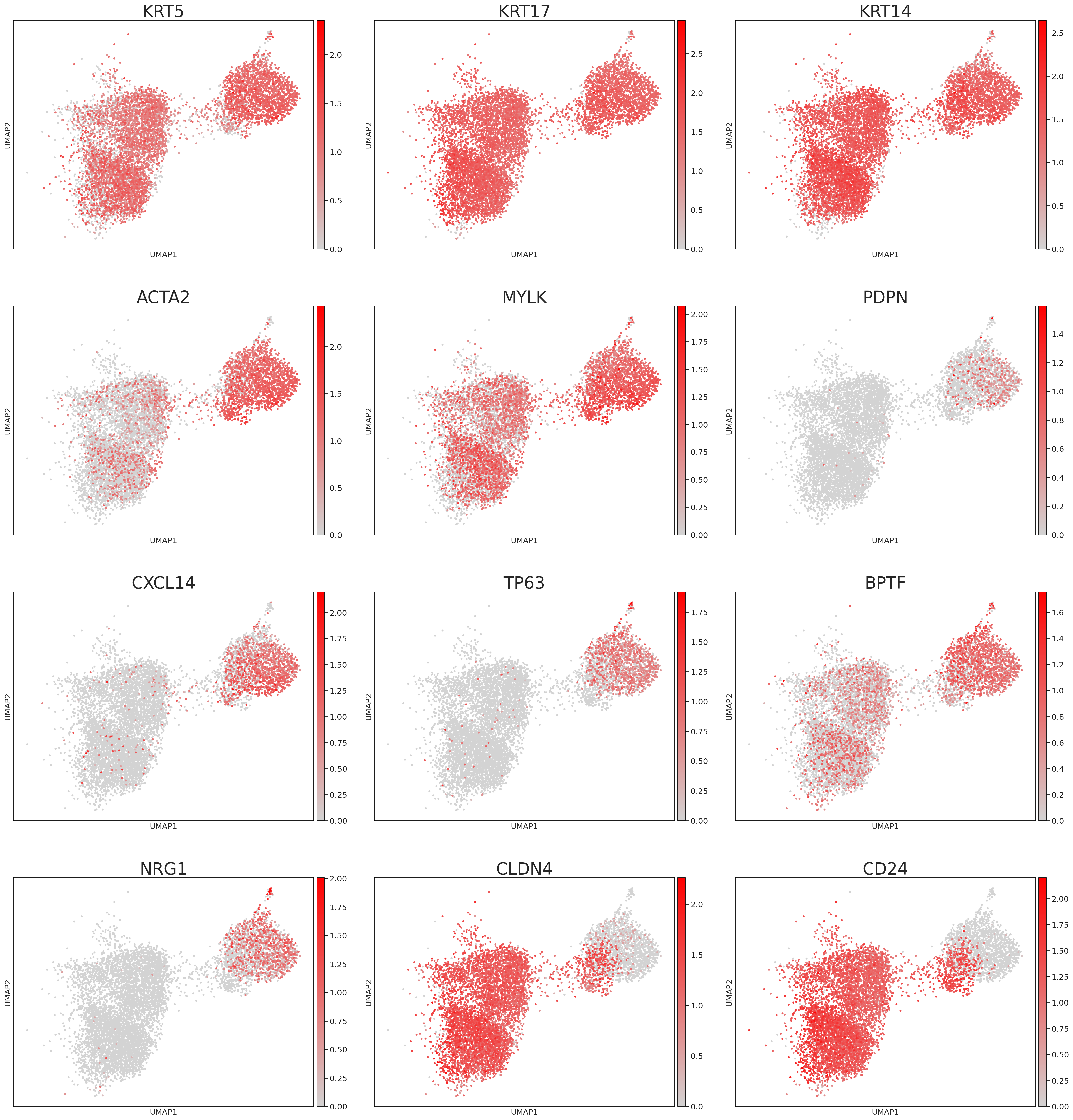

Supplement: Supplementary file 6 — Fig. S5. Uniform manifold approximation and projections (UMAPs) of proliferative basal cancer cells (PBCs), nonproliferative basal cancer cells (NPBCs), and myoepithelial (MYO) cells for MYO markers. This multipanel figure displays the normalized expression level of selected genes for MYO cell characterization. Each subpanel represents a different gene, with color intensity ranging from gray (low or no expression) to bright red (high expression), as indicated by the common color scale bar on the right of each plot. [file FEB2-599-3124-s005.tiff]

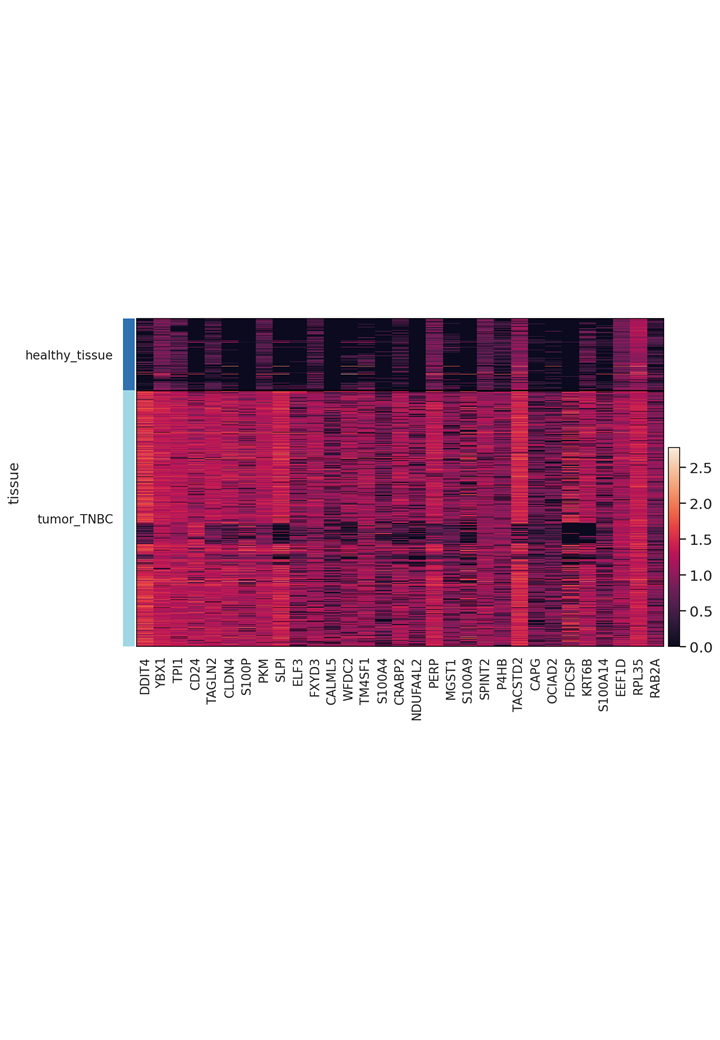

Supplement: Supplementary file 7 — Fig. S6. Differential gene expression of the top 30 markers upregulated in myoepithelial (MYO)‐like cancer cells. This heatmap displays the normalized expression levels of the top 30 genes identified as markers of aneuploid MYO‐like cancer cells. Each row represents a single cell from either healthy breast tissue or triple‐negative breast cancer (TNBC) patient tumors, as indicated by the blue (healthy_tissue) and light blue (tumor_TNBC) sidebar annotation on the Y‐axis. The X‐axis lists the top 30 differentially upregulated genes. The color intensity within the heatmap reflects gene expression levels, with darker shades (black/dark red) indicating lower expression and brighter shades (pink/yellow/white) indicating higher expression, as shown by the color scale bar. [file FEB2-599-3124-s006.tif]

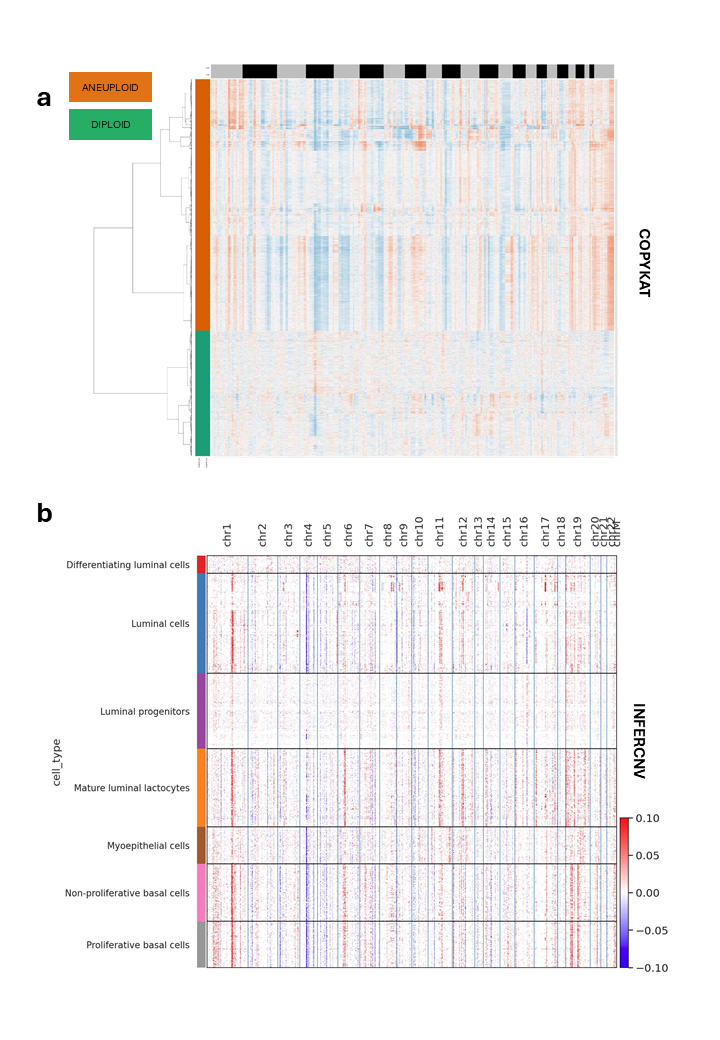

Supplement: Supplementary file 8 — Fig. S7. Copy number variation (CNV) heatmap of epithelial cells. The two heatmaps display inferred whole‐genome CNVs for individual epithelial cells using the CopyKAT (a) and InferCNVpy (b) software tools. Chromosomes are ordered sequentially along the X‐axis, and each row on the Y‐axis represents a single cell. In the heatmaps, color intensity reflects the genome copy number status, where blue indicates deletions and red indicates chromosomal amplifications. In a, cells grouped by the orange sidebar are computationally inferred as aneuploid (malignant), while those grouped by the green sidebar are inferred as diploid (nonmalignant/normal). In b, cells are grouped by the colored sidebar according to their annotation. [file FEB2-599-3124-s001.tif]

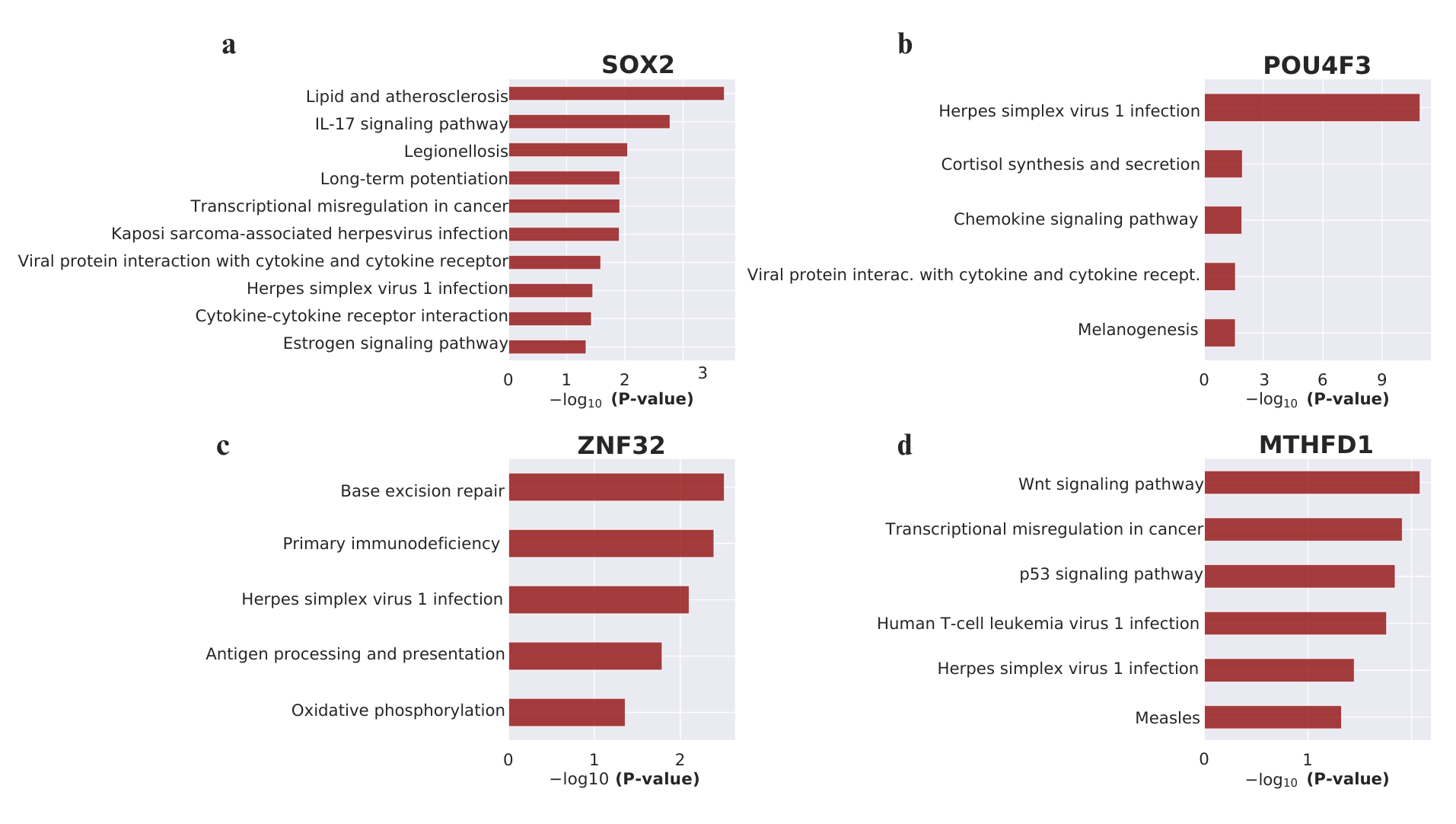

Supplement: Supplementary file 9 — Fig. S8. Enrichment plots of common regulons. Bar plots of pathway enrichment analysis. The X axis represents the significance as –log10(P‐value), while in the Y axis, the different pathways are listed. [file FEB2-599-3124-s009.tiff]

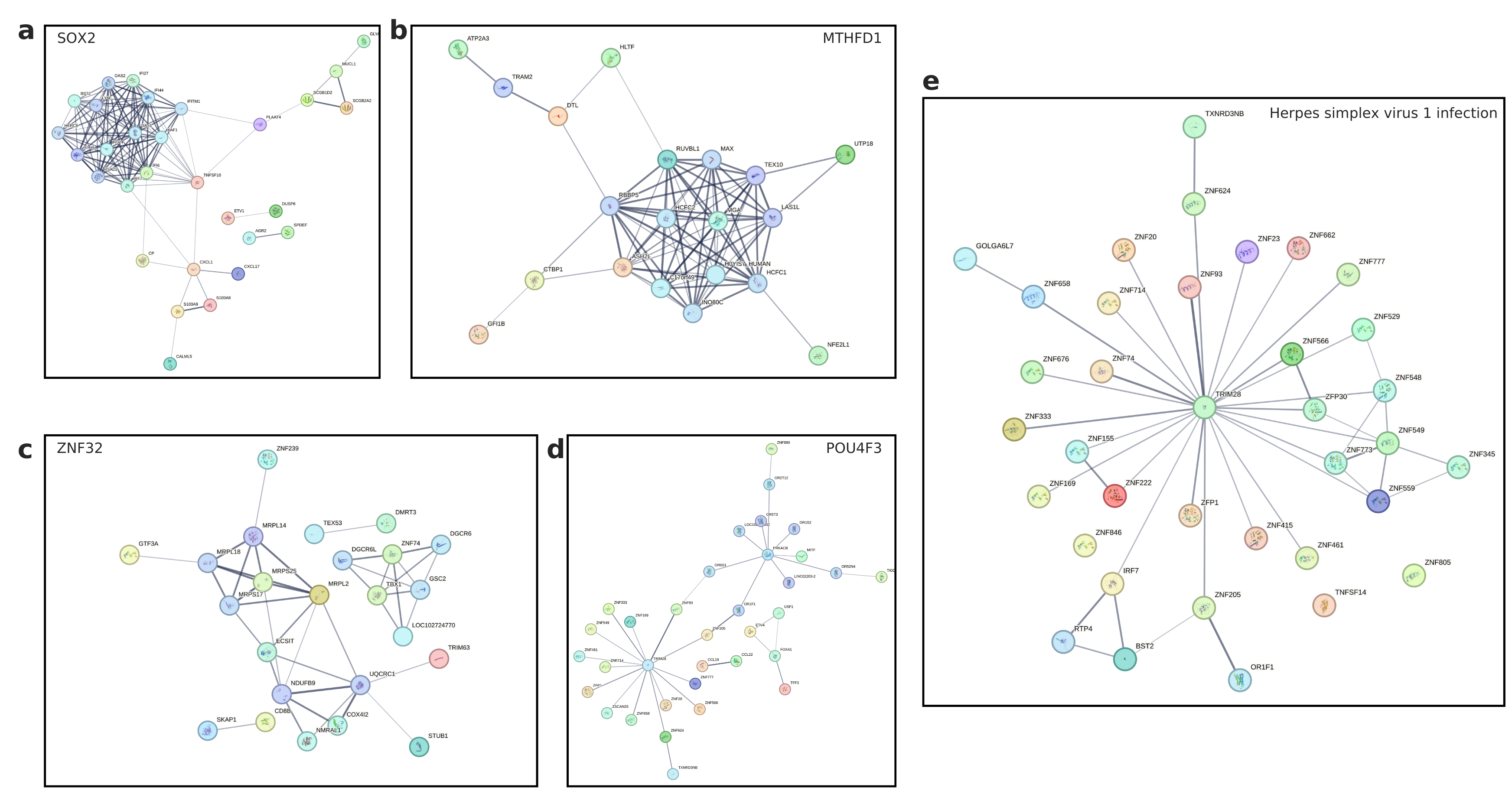

Supplement: Supplementary file 10 — Fig. S9. Gene networks of top 50 targets of the four uniquely active regulon (SOX2, MTHFD1, ZNF32, and POU4F3). The nodes in the networks represent the input genes (top 50 targets of each regulon) and their first shell of 10 interactors based on scientific evidence. The thickness of the lines represents the strength of the evidence of interactions between two nodes, setting medium confidence as threshold. Made with https://string‐db.org/ accessed on 25 March 2025. [file FEB2-599-3124-s008.tiff]

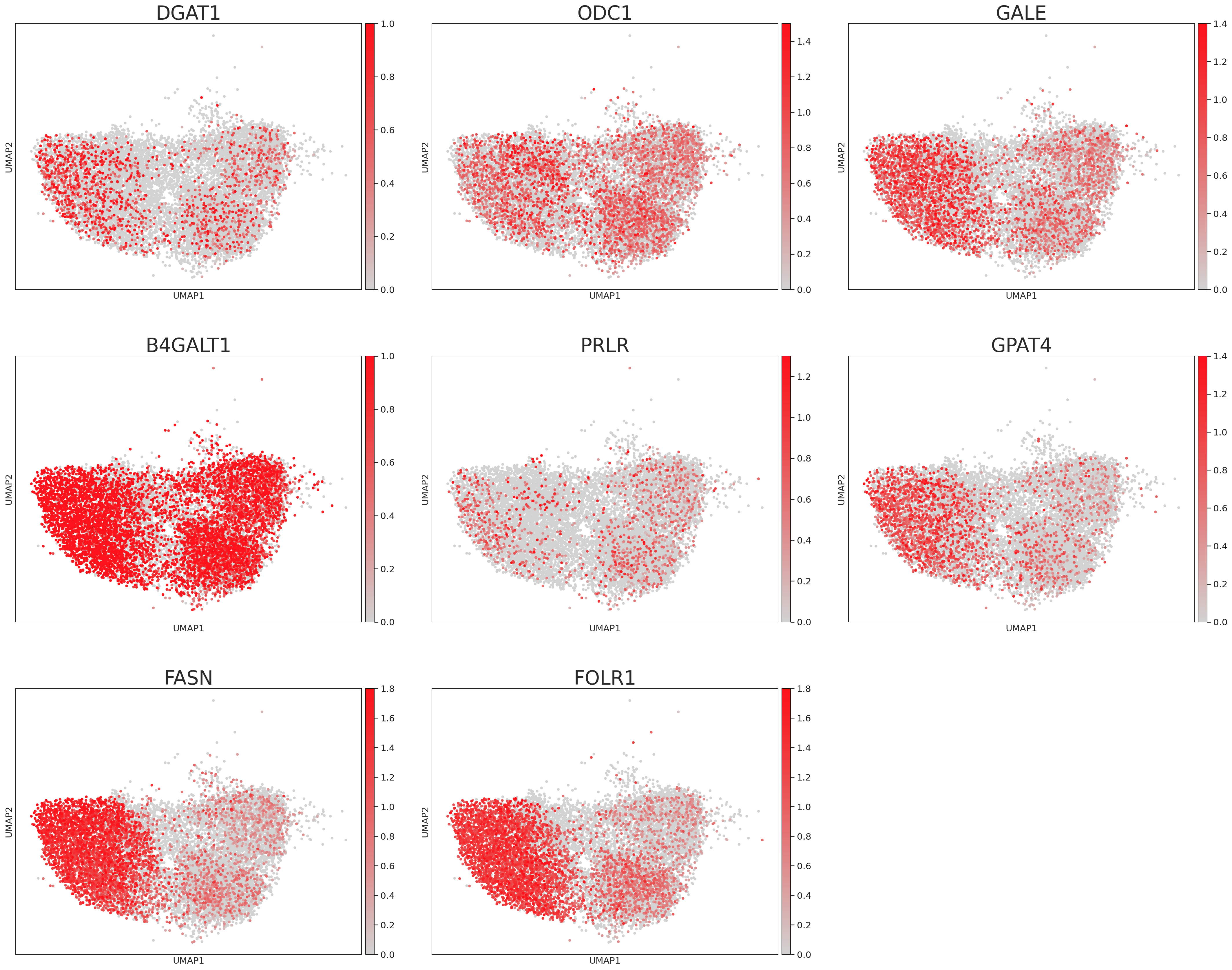

Supplement: Supplementary file 11 — Fig. S10. Expression of genes shared by triple‐negative breast cancer cells (TNBCs) and mature luminal lactocytes (MLLs) and upregulated during lactation. Each panel shows the expression of the indicated gene in the two cell types. [file FEB2-599-3124-s012.tiff]

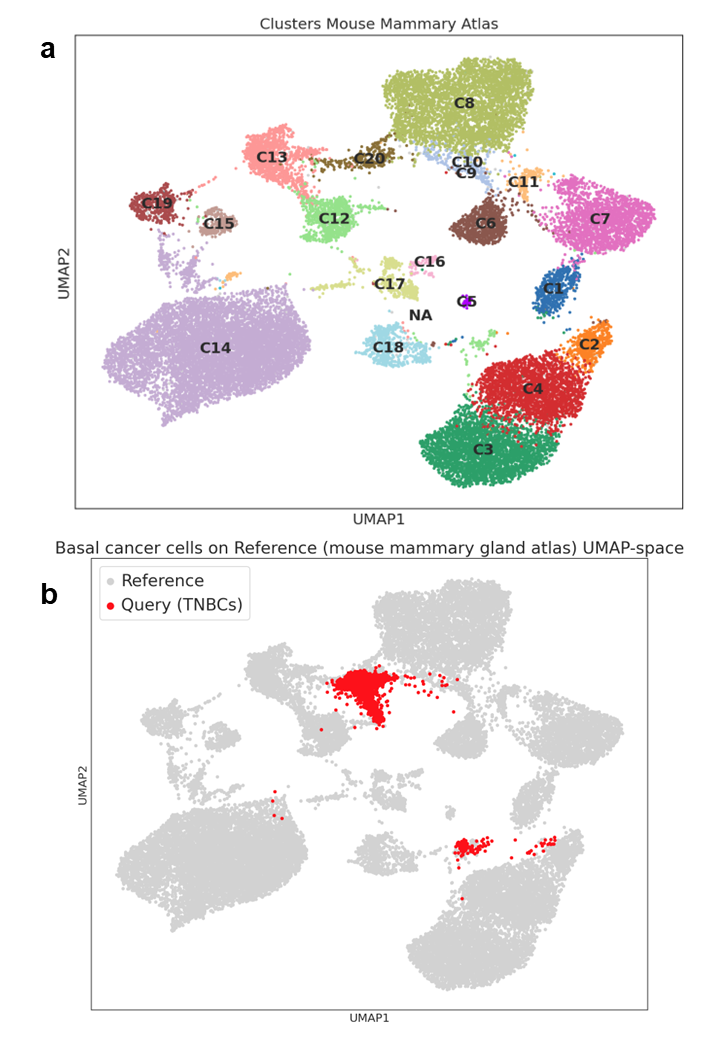

Supplement: Supplementary file 12 — Fig. S11. Reference mapping of query cells onto the mammary gland development atlas. (a) the uniform manifold approximation and projection (UMAP) of the reference dataset (mouse mammary gland development atlas) where cells are colored according to their annotated cluster. (b) the transcriptomic profiles of the query cells (nonproliferative basal cancer cells, NPBC; proliferative basal cancer cells, PBC) were mapped onto the UMAP space defined by the reference atlas using reference mapping (the scanpy ingest integrated tool). Reference cells are shown in light gray to provide contextual embedding, while query cells are highlighted in red. [file FEB2-599-3124-s014.tif]

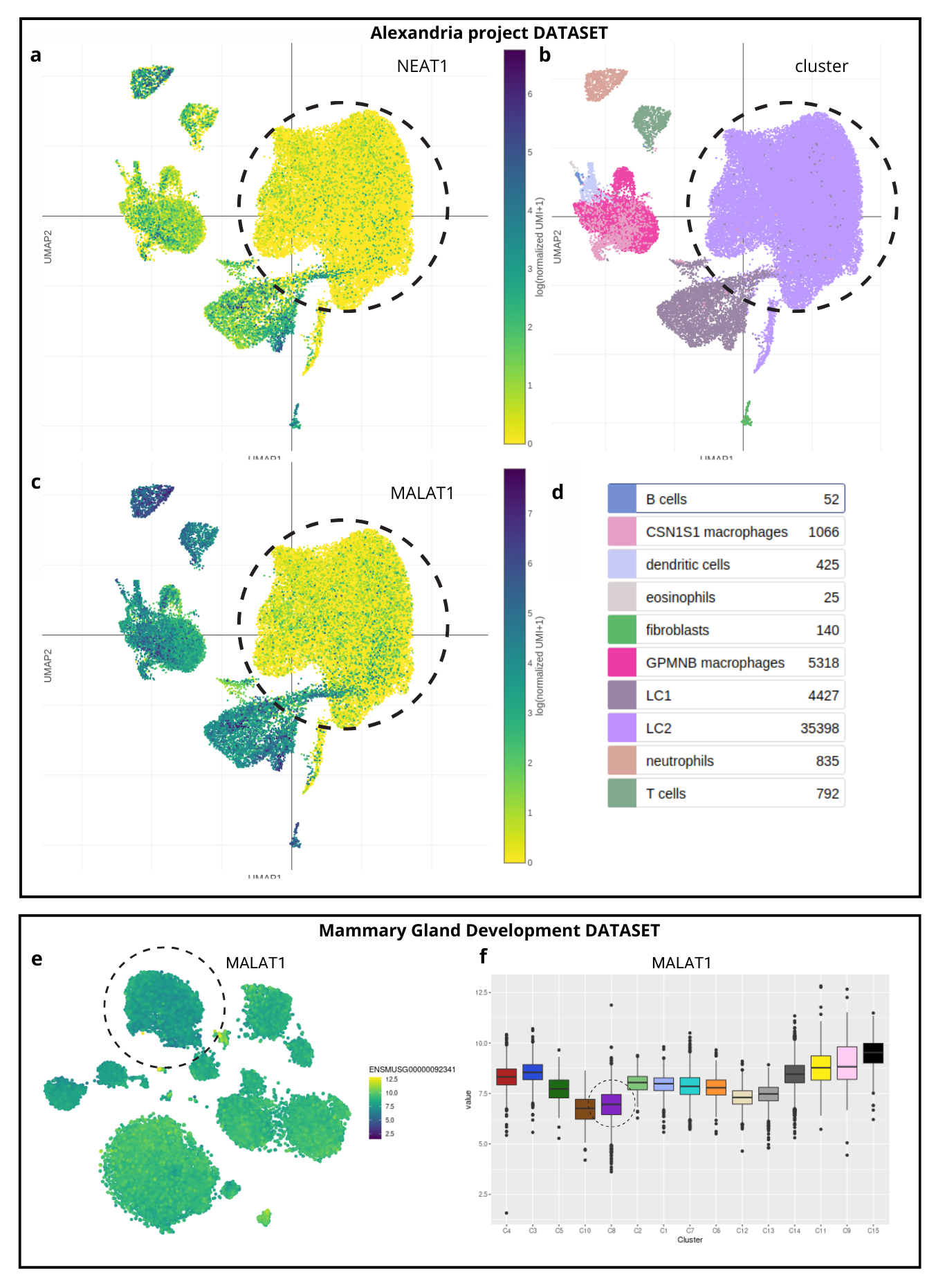

Supplement: Supplementary file 13 — Fig. S12. MALAT1 and NEAT1 validation. On the top panel, representing the Alexandria project data (https://singlecell.broadinstitute.org/single_cell?scpbr=the‐alexandria‐project), in a and c, the uniform manifold approximation and projections (UMAP) of MALAT1 and NEAT1 expression respectively; in b, the UMAP representation of single‐cell clusters annotated by cell types; in d, the cell labels corresponding to the cell types of the different clusters of b. The data and graphs were obtained using the BROAD institute Single Cell Portal (https://singlecell.broadinstitute.org/single_cell, last accessed on 03/03/2025). On the bottom panel, in e, the t‐distributed stochastic neighbor embedding (t‐SNE) representation of Malat1 (ENSMUSG00000092341) expression in murine mammary tissue; in f, the corresponding boxplot where in the X axis, the different clusters are listed, while Y is the expression value. The black dotted circles highlight the clusters of interest representing the milk‐producing cells (differentiated alveolar cells or mature luminal lactocytes). Graphs in e and f have been obtained using the RShiny app from the Marioni Lab (https://marionilab.cruk.cam.ac.uk/mammaryGland/). [file FEB2-599-3124-s002.tiff]
